# Genetic Loci for Retinal Arteriolar Microcirculation

**DOI:** 10.1371/journal.pone.0065804

**Published:** 2013-06-12

**Authors:** Xueling Sim, Richard A. Jensen, M. Kamran Ikram, Mary Frances Cotch, Xiaohui Li, Stuart MacGregor, Jing Xie, Albert Vernon Smith, Eric Boerwinkle, Paul Mitchell, Ronald Klein, Barbara E. K. Klein, Nicole L. Glazer, Thomas Lumley, Barbara McKnight, Bruce M. Psaty, Paulus T. V. M. de Jong, Albert Hofman, Fernando Rivadeneira, Andre G. Uitterlinden, Cornelia M. van Duijn, Thor Aspelund, Gudny Eiriksdottir, Tamara B. Harris, Fridbert Jonasson, Lenore J. Launer, The Wellcome Trust Case Control Consortium 2, John Attia, Paul N. Baird, Stephen Harrap, Elizabeth G. Holliday, Michael Inouye, Elena Rochtchina, Rodney J. Scott, Ananth Viswanathan, Global BPGen Consortium, Guo Li, Nicholas L. Smith, Kerri L. Wiggins, Jane Z. Kuo, Kent D. Taylor, Alex W. Hewitt, Nicholas G. Martin, Grant W. Montgomery, Cong Sun, Terri L. Young, David A. Mackey, Natalie R. van Zuydam, Alex S. F. Doney, Colin N. A. Palmer, Andrew D. Morris, Jerome I. Rotter, E. Shyong Tai, Vilmundur Gudnason, Johannes R. Vingerling, David S. Siscovick, Jie Jin Wang, Tien Y. Wong

**Affiliations:** 1 Center for Statistical Genetics, University of Michigan, Ann Arbor, Michigan, United States of America; 2 Cardiovascular Health Research Unit, University of Washington, Seattle, Washington, United States of America; 3 Department of Epidemiology, University of Washington, Seattle, Washington, United States of America; 4 Singapore Eye Research Institute, Singapore National Eye Centre, Singapore, Singapore; 5 Department of Ophthalmology, Erasmus Medical Center, Rotterdam, The Netherlands; 6 Division of Epidemiology and Clinical Applications, National Eye Institute, Intramural Research Program, National Institutes of Health, Bethesda, Maryland, United States of America; 7 Medical Genetics Institute, Cedars-Sinai Medical Center, Los Angeles, California, United States of America; 8 Genetics and Population Health, Queensland Institute of Medical Research, Brisbane, Queensland, Australia; 9 Centre for Eye Research Australia, University of Melbourne, Royal Victorian Eye and Ear Hospital, Melbourne, Victoria, Australia; 10 Icelandic Heart Association, Kopavogur Capital Region, Iceland; 11 Department of Medicine, University of Iceland, Reykjavik, Iceland; 12 Human Genetics Center and Institute of Molecular Medicine, University of Texas Health Science Center at Houston, Houston, Texas, United States of America; 13 Centre for Vision Research, Department of Ophthalmology and the Westmead Millennium Institute, University of Sydney, Sydney, New South Wales, Australia; 14 Department of Ophthalmology and Visual Sciences, School of Medicine and Public Health, University of Wisconsin, Madison, Wisconsin, United States of America; 15 Department of Medicine, University of Washington, Seattle, Washington, United States of America; 16 Department of Medicine, Boston University, Boston, Massachusetts, United States of America; 17 Department of Biostatistics, University of Washington, Seattle, Washington, United States of America; 18 Department of Statistics, University of Auckland, Auckland, New Zealand; 19 Department of Health Services, University of Washington, Seattle, Washington, United States of America; 20 Group Health Research Institute, Group Health Cooperative, Seattle, Washington, United States of America; 21 Department of Clinical and Molecular Ophthalmogenetics, The Netherlands Institute of Neuroscience, Amsterdam, The Netherlands; 22 Department of Ophthalmology, Academic Medical Center, Amsterdam, The Netherlands; 23 Department of Epidemiology, Erasmus Medical Center, Rotterdam, The Netherlands; 24 Department of Internal Medicine, Erasmus Medical Center, Rotterdam, The Netherlands; 25 Department of Clinical Chemistry, Erasmus Medical Center, Rotterdam, The Netherlands; 26 Laboratory of Epidemiology, Demography, and Biometry, National Institute on Aging, Intramural Research Program, National Institutes of Health, Bethesda, Maryland, United States of America; 27 Department of Ophthalmology, University of Iceland, Reykjavik, Iceland; 28 Department of Ophthalmology, Landspitalinn University Hospital, Reykjavik, Iceland; 29 School of Medicine and Public Health, University of Newcastle, Newcastle, New South Wales, Australia; 30 Department of Medicine, John Hunter Hospital and Hunter Medical Research Institute, Newcastle, New South Wales, Australia; 31 Department of Physiology, University of Melbourne, Melbourne, Victoria, Australia; 32 Immunology Division, Walter and Eliza Hall Institute of Medical Research, Victoria, Australia; 33 Department of Medical Biology, University of Melbourne, Victoria, Australia; 34 School of Biomedical Sciences, University of Newcastle, Newcastle, New South Wales, Australia; 35 National Institutes of Health Research (NIHR) Biomedical Research Centre for Ophthalmology, Moorfields Eye Hospital, London, United Kingdom; 36 University College London Institute of Ophthalmology, London, United Kingdom; 37 Seattle Epidemiologic Research and Information Center, Veterans Affairs Office of Research and Development, Seattle, Washington, United States of America; 38 Murdoch Children's Research Institute, Royal Children's Hospital, Melbourne, Victoria, Australia; 39 Center for Human Genetics, Duke University Medical Center, Durham, North Carolina, United States of America; 40 Lions Eye Institute, University of Western Australia, Centre for Ophthalmology and Visual Science, Perth, Western Australia, Australia; 41 Medical Research Institute, University of Dundee, Dundee, Scotland, United Kingdom; 42 Department of Medicine, National University of Singapore, Singapore, Singapore; 43 Saw Swee Hock School of Public Health, National University of Singapore, Singapore, Singapore; 44 Duke-National University of Singapore Graduate Medical School, Singapore, Singapore; 45 Department of Ophthalmology, National University of Singapore, Singapore, Singapore; University of Birmingham, United Kingdom

## Abstract

Narrow arterioles in the retina have been shown to predict hypertension as well as other vascular diseases, likely through an increase in the peripheral resistance of the microcirculatory flow. In this study, we performed a genome-wide association study in 18,722 unrelated individuals of European ancestry from the Cohorts for Heart and Aging Research in Genomic Epidemiology consortium and the Blue Mountain Eye Study, to identify genetic determinants associated with variations in retinal arteriolar caliber. Retinal vascular calibers were measured on digitized retinal photographs using a standardized protocol. One variant (rs2194025 on chromosome 5q14 near the myocyte enhancer factor 2C *MEF2C* gene) was associated with retinal arteriolar caliber in the meta-analysis of the discovery cohorts at genome-wide significance of *P*-value <5×10^−8^. This variant was replicated in an additional 3,939 individuals of European ancestry from the Australian Twins Study and Multi-Ethnic Study of Atherosclerosis (rs2194025, *P*-value = 2.11×10^−12^ in combined meta-analysis of discovery and replication cohorts). In independent studies of modest sample sizes, no significant association was found between this variant and clinical outcomes including coronary artery disease, stroke, myocardial infarction or hypertension. In conclusion, we found one novel loci which underlie genetic variation in microvasculature which may be relevant to vascular disease. The relevance of these findings to clinical outcomes remains to be determined.

## Introduction

The retinal microcirculation provides a unique window for non-invasive visualization of the human microcirculation *in vivo*. Retinal vascular caliber has been shown to predict cardiovascular diseases [1], [2], [3], [4]. Narrow retinal arterioles have been documented to be a risk factor for subsequent development of hypertension [5], [6], [7], [8], diabetes mellitus [9], [10], [11], stroke [12], coronary heart disease [3], [4], [13] and cerebral small vessel disease [14], [15], [16]. The most consistent evidence is that narrow retinal arteriolar caliber, reflecting widespread peripheral vasoconstriction and arteriolosclerosis, precedes clinically manifest hypertension and, therefore, is a pre-clinical marker for subsequent elevation of blood pressure [17], [18].

Recent studies suggest that genetic factors may influence retinal vascular caliber [19], [20], [21], [22]. The identification of genetic determinants of retinal vascular caliber may provide further insights into the relationship between retinal vessels and cardiovascular diseases. We recently reported on four novel loci associated with retinal venular caliber in 15,358 subjects from the Cohorts for Heart and Aging research in Genomic Epidemiology (CHARGE) consortium. However, there was no genome-wide significant association between any single nucleotide polymorphism (SNP) and retinal arteriolar caliber [23].

In this study, we extended our discovery phase by including the Blue Mountains Eye Study (BMES) [24], which measured retinal vascular caliber and recently completed genome-wide marker genotyping of 2,430 participants. We replicated our findings in the European white populations of the Multi-Ethnic Study of Atherosclerosis (MESA) [25] and the Australian Twins Study [26]. Finally, we sought to determine whether any of the loci are also associated with major macrovascular disease outcomes in independent cohorts of European ancestry, including hypertension in the Global Blood Pressure Genetics (Global BPgen) [27], coronary artery disease (CAD) in the Wellcome Trust Case Control Consortium (WTCCC) [28], myocardial infarction and stroke in the Heart and Vascular Study (HVH) [29], [30] and incident/prevalent coronary artery disease and incident ischemic stroke in the Genetics of Diabetes Audit and Research in Tayside Scotland (GoDARTS) [31].

## Results

### Study Cohorts

The discovery phase examined data from 18,722 individuals of European ancestry from the CHARGE consortium [32] and BMES [24]. The sample size for the replication phase was 3,939 individuals, also of European ancestry. Characteristics of the cohorts in both the discovery and replication phases are presented in **Table 1**.

**Table 1:** Baseline characteristics of the discovery and replication cohorts.

	Discovery cohorts					Replication cohorts	
	AGES	ARIC	CHS	RS	BMES	Australian Twins	MESA
N	2,949	7,260	1,263	4,820	2,430	1,769	2,170
Age (years)	76.2 (5.4)	59.9 (5.6)	78.4 (4.2)	68.0 (8.2)	66.8 (9.0)	22.0 (11.9)	62.2 (10.0)
	[66–94]	[50–71]	[72–95]	[55–99]	[49–96]	[5–90]	[44–84]
Proportion female (%)	57.5	53.5	63.0	59.0	56.9	56.0	49.0
CRAE (µm)	139.7 (13.4)	135.1 (12.8)	138.8 (14.2)	150.0 (14.4)	160.0 (15.4)	164.3 (13.6)	142.7 (14.3)
	[74.0–221.4]	[72.6–187.2]	[77.6–191.0]	[98.5–235.4]	[91.4, 210.7]	[83.6–205.2]	[83.3–219.2]
CRVE (µm)	202.0 (19.5)	199.5 (19.1)	196.5 (19.2)	226.0 (20.1)	224.6 (20.2)	248.0 (19.0)	206.5 (21.1)
	[123.8–273.0]	[129.3–304.1]	[142.5–271.7]	[162.5–324.3]	[150.0–311.7]	[130.5–325.7]	[123.6–289.9]
Body mass index (kg/m^2^)	27.1 (4.4)	28.0 (5.2)	26.8 (4.3)	26.3 (3.7)	27.6 (4.7)	NA	27.7 (5.0)
	[14.8–48.5]	[14.2–59.1]	[15.6–46.7]	[14.2–50.7]	[16.5–49.2]		[16.9–49.0]
Systolic blood pressure (mmHg)	142.5 (20.2)	122.4 (18.0)	134.3 (20.4)	138.5 (22.1)	146.0 (21.4)	NA	122.6 (19.9)
	[92.0–253.0]	[63.0–226.0]	[82.0–241.0]	[74.0–250.0]	[90.0–235.0]		[75.0–208.5]
Diastolic blood pressure (mmHg)	74.1 (20.2)	70.74 (10.0)	67.9 (10.8)	73.7 (11.4)	84.8 (10.2)	NA	70.1 (9.9)
	[92.0–253.0]	[32.0–114.0]	[15.0–110.0]	[24.0–139.0]	[50.0–120.0]		[41.0–108.0]
Hypertension (%)	80.6	39.3	48.8	42.3	50.9	3.2	37.5
Diabetes mellitus (%)	11.4	11.9	12.3	10.0	10.3	1.0	5.4
Current smoker (%)	12.5	17.3	6.1	23.6	9.8	11.0	11.2

AGES: Age Gene/Environment Susceptibility – Reykjavik Study, ARIC: Atherosclerosis Risk in Communities Study, CHS: Cardiovascular Health Study, RS: Rotterdam Study, BMES: Blue Mountains Eye Study. MESA: Multi-Ethnic Study of Atherosclerosis. Mean (standard deviation) [range] are given for continuous variables. Percentages are given for categorical variables.

### Meta-analysis of Discovery Cohorts

**Figure 1:**
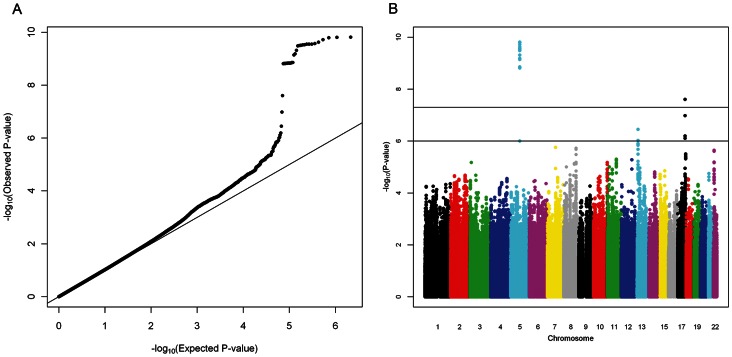
A) QQ-plot of –log_10_(observed *P*-values) against –log_10_(expected *P*-values) and B) Manhattan plot of –log_10_ transformed *P*-values of retinal arteriolar caliber against their physical position.

**Figure 2:**
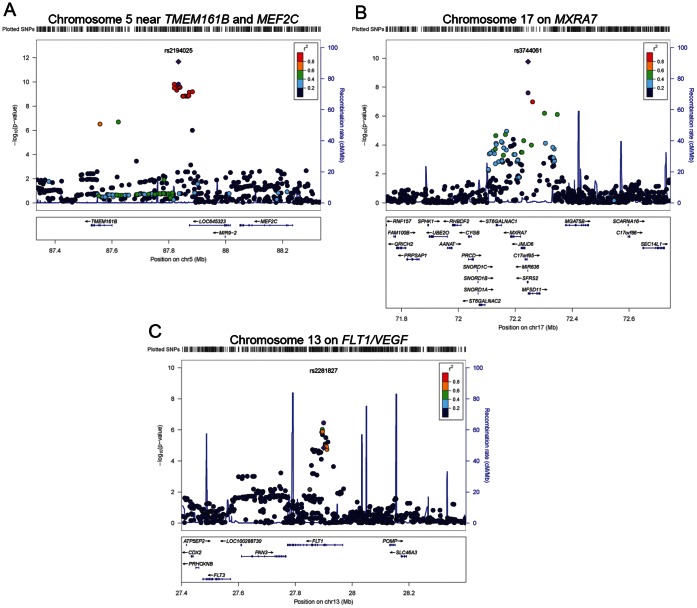
Regional association plots at the two loci that exhibit genome-wide significance at discovery stage and one locus that showed suggestive evidence of association at *P*-value <10^−6^. A) Chromosome 5 near *TMEM161B* and *MEF2C*, B) Chromosome 17 on *SFRS2* and C) Chromosome 13 on *FLT*. In each regional plot, the index SNP is represented by a purple circle for the meta-analysis of the five discovery studies and a purple diamond for meta-analysis of discovery and replication studies. The remaining SNPs are colour coded according to pairwise linkage disequilibrium (LD) with the index SNP on a scale of r^2^ from 0 (blue) to 1 (red). Estimated recombination rates reflect the local LD structure in the 500 kb buffer around the index SNP and plotted based on values on Hapmap II CEU. Data for gene annotations are obtained from the RefSeq track of the UCSC Gene Browser (See LocusZoom http://csg.sph.umich.edu/locuszoom/ more details).

A total of 2,137,729 genotyped or imputed SNPs post quality control were common to all five cohorts. The QQ-plot (**Figure 1A**) showed departure from the line of identity around *P*-values <1.0×10^−3^, with an overall genomic inflation factor of 1.03. The Manhattan plot of minus log-transformed *P*-values for each SNP against its physical position showed two loci reaching genome-wide significance, one on chromosome 5 and the other on chromosome 17 (**Figure 1B**). These included a cluster of 31 SNPs in high linkage disequilibrium on chromosome 5 between the genes *TMEM161B* (transmembrane protein 161B) and *MEF2C* (myocyte enhancer factor 2C) and 2 SNPs on chromosome 17, with a number of genes spanning the region including *SFRS2* (serine/arginine-rich splicing factor 2), *MFSD11* (major facilitator superfamily domain containing 11), *JMJD6* (jumonji domain containing 6) and *MXRA7* (matrix-remodelling associated 7) (**Figure 2**). A third locus on chromosome 13 exhibited suggestive evidence of an association with retinal arteriolar caliber at *P*-value <10^−6^, on *FLT1* (fms-related tyrosine kinase 1, also known as the vascular endothelial growth factor) (**Table 2**). The coded allele for each SNP was presented as the allele that decreased arteriolar caliber; and we referred to this as the effective allele. A second model was fitted for the top index SNPs in each discovery cohort, with additional adjustment for hypertension and diabetes status. The association of the three index SNPs with retinal arteriolar caliber remained unchanged (**Table S1**). Collectively, these variants only explained 0.52 to 1.25% of the overall variance in retinal arteriolar caliber in each of the discovery cohorts. As these index SNPs were imputed SNPs, we also presented the next best index SNPs directly observed in **Table S2**.

**Table 2:** Association of index SNP with retinal arteriolar caliber at three top loci for each discovery cohort and meta-analysis.

SNP (chr: position)	Ref/Effective allele (+)	Cohort	Eff allele freq (G/I)	Beta (SE)	*P*-value	Genes of interest	# SNPs *P*-value <10^−6^
rs2194025 (5: 87833992)	C/G	AGES	0.95 (I: 0.95)	−1.32 (0.80)	1.01×10^−1^	*TMEM16B*	31
		ARIC	0.90 (I: 0.99)	−1.28 (0.37)	4.83×10^−4^	*MEF2C*	
		CHS	0.91 (I: 0.96)	−3.20 (1.11)	3.70×10^−3^		
		RS	0.90 (I: 0.99)	−1.40 (0.49)	4.15×10^−3^		
		BMES	0.90 (I: 0.99)	−2.90 (0.73)	6.69×10^−5^		
		**Discovery cohorts combined**	**0.91**	**−1.60 (0.25)**	**1.53×10** ^−**10**^		
		**Cohort**	**Eff allele freq (G/I)**	**Beta (SE)**	***P*** **-value**	**Genes of interest**	**# SNPs ** ***P*** **-value <10^−6^**
rs3744061 (17: 72244998)	A/G	AGES	0.46 (I: 0.92)	−0.95 (0.36)	7.89×10^−3^	*SFRS2*	4
		ARIC	0.44 (I: 0.95)	−1.04 (0.22)	2.31×10^−6^	*MFSD11*	
		CHS	0.43 (I: 0.88)	−1.15 (0.63)	6.89×10^−2^	*JMJD6*	
		RS	0.42 (I: 0.95)	−0.29 (0.31)	3.48×10^−1^	*MXRA7*	
		BMES	0.45 (I: 0.95)	−0.63 (0.45)	1.55×10^−1^		
		**Discovery cohorts combined**	**0.44**	**−0.82 (0.15)**	**2.49×10** ^−**8**^		
		**Cohort**	**Eff allele freq (G/I)**	**Beta (SE)**	***P*** **-value**	**Genes of interest**	**# SNPs ** ***P*** **-value <10^−6^**
rs2281827 (13: 27899721)	T/C	AGES	0.78 (I: 0.89)	−1.27 (0.45)	4.96×10^−3^	*FLT1*	2
		ARIC	0.77 (I: 0.92)	−0.42 (0.26)	1.09×10^−1^		
		CHS	0.73 (I: 0.81)	−1.04 (0.68)	1.26×10^−1^		
		RS	0.78 (I: 0.88)	−1.63 (0.38)	1.66×10^−5^		
		BMES	0.76 (I: 0.89)	−0.84 (0.54)	1.22×10^−1^		
		**Discovery cohorts combined**	**0.77**	**−0.90 (0.18)**	**3.55×10** ^−**7**^		

AGES: Age Gene/Environment Susceptibility – Reykjavik Study, ARIC: Atherosclerosis Risk in Communities Study, CHS: Cardiovascular Health Study, RS: Rotterdam Study, BMES: Blue Mountains Eye Study.

The allele that decreases retinal arteriolar caliber is presented as the effective allele.

(G/I): Indicates if the SNP is directly genotyped (G) or imputed (I). If the SNP is imputed, the imputation quality is included.

### Replication in Independent Cohorts

**Table 3** shows results from each of the two replication cohorts, MESA and Australian Twins Study, for the three index SNPs on chromosomes 5, 13 and 17 that were taken forward for replication from the discovery phase. Minor allele frequencies in the replication cohorts were very similar to those in the discovery cohorts. Of the three variants that were taken forward for replication, two SNPs replicated in the combined analyses of the replication cohorts. These included rs2194025 on chromosome 5 (*P*-value = 3.74×10^−3^) and rs3744061 on chromosome 17 (*P*-value = 1.51×10^−3^). In both instances, the directions of effect in the replication cohorts were consistent with the directions of effect observed in the discovery phase (**Table 3**). Index SNP rs2194025 on chromosome 5 yielded an overall *P*-value of 2.11×10^−12^ in the meta-analysis including both discovery and replication cohorts. Each additional copy of the effective allele was associated with a decrease of 1.6 µm in the mean retinal arteriolar caliber. Similar evidence of association was seen for rs2194026, a directly genotyped SNP on chromosome 5. For SNP rs3744061 on chromosome 17 locus, each additional copy of the effective allele was associated with a decrease of 0.86 µm in mean arteriolar caliber (*P*-value = 1.74×10^−10^); however, the next best directly genotyped SNP rs9916811 did not replicate (*P*-value for discovery cohorts = 1.05×10^−8^; *P*-value for discovery and replication cohorts combined by meta-analysis = 1.77×10^−5^). SNP rs2281827 on chromosome 13 also did not replicate consistently; and the overall *P*-value in the meta-analysis including both the discovery and replication cohorts did not reach genome-wide significance. The regional association plots are shown in **Figure 2** for all three loci.

**Table 3:** Results for retinal arteriolar caliber for discovery cohort meta-analysis and replication cohorts.

SNP (chr: position)	Ref/Effectiveallele (+)	Cohort	Eff allelefreq	Beta (SE)	*P*-value	Genes of interest
rs2194025 (5: 87833992)	C/G	Discovery cohorts combined	0.91	−1.60 (0.25)	1.53×10^−10^	*TMEM16B*
		MESA Whites	0.91	−2.03 (0.76)	7.00×10^−3^	*MEF2C*
		Australian Twins	0.91	−1.14 (0.83)	1.68×10^−1^	
		Replication cohorts combined	0.91	−1.62 (0.56)	3.74×10^−3^	
		**Discovery+Replication cohorts combined**	**0.91**	−**1.60 (0.23)**	**2.11×10** ^−**12**^	
		**Cohort**	**Eff allele freq**	**Beta (SE)**	***P*** **-value**	**Genes of interest**
rs3744061 (17: 72244998)	A/G	Discovery cohorts combined	0.44	−0.82 (0.15)	2.49×10^−8^	*SFRS2*
		MESA Whites	0.44	−0.44 (0.45)	3.20×10^−1^	*MFSD11*
		Australian Twins	0.45	−1.75 (0.49)	3.01×10^−4^	*JMJD6*
		Replication cohorts combined	0.45	−1.04 (0.33)	1.51×10^−3^	*MXRA7*
		**Discovery+Replication cohorts combined**	**0.44**	−**0.86 (0.13)**	**1.74×10** ^−**10**^	
		**Cohort**	**Eff allele freq**	**Beta (SE)**	***P*** **-value**	**Genes of interest**
rs2281827 (13: 27899721)	T/C	Discovery cohorts combined	0.77	−0.90 (0.18)	3.55×10^−7^	*FLT1*
		MESA Whites	0.75	−0.15 (0.51)	7.70×10^−1^	
		Australian Twins	0.76	0.74 (0.57)	1.92×10^−1^	
		Replication cohorts combined	0.75	0.25 (0.38)	5.12×10^−1^	
		**Discovery+Replication cohorts combined**	**0.76**	−**0.70 (0.16)**	**1.44×10** ^−**5**^	

SE: standard error, OR: odds ratio, MESA: Multi-Ethnic Study of Atherosclerosis.

The allele that decreases retinal arteriolar caliber is presented as the effective allele (refer to Table 2).

### Conditional Analyses

**Figure 3:**
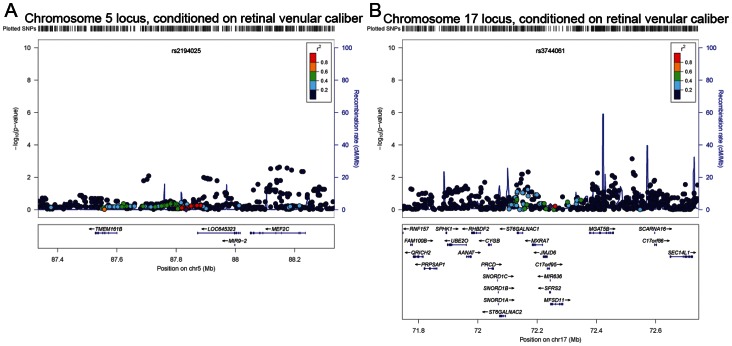
Regional association plots for retinal arteriolar caliber conditioned on retinal venular caliber for A) Chromosome 5 near *TMEM161B* and *MEF2C* and B) Chromosome 17 on *SFRS2*. All regional plots are centered on the index SNPs, with a 500 kb buffer on both sides of the index SNP.

**Figure 4:**
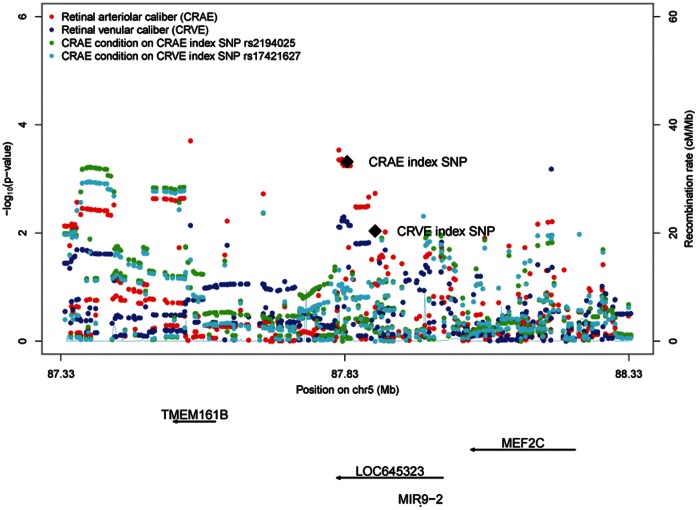
Regional association plot at chromosome 5 locus using ARIC data, showing *P*-values for SNPs for retinal arteriolar caliber (CRAE), retinal venular caliber (CRVE), retinal arteriolar caliber conditioned on CRAE index SNP rs2194025 and retinal arteriolar caliber conditioned on CRVE implicated SNP rs17421627.

The coefficient of correlation between arteriolar and venular caliber is approximately 0.6 [33], [34]. To account for this relationship, additional regression models were fitted for retinal arteriolar caliber conditioning on retinal venular caliber. These conditional analyses effectively removed any association with the SNPs on chromosomes 5 and 17, although it is possible that there is potential over-adjustment by including retinal venular caliber as a covariate (**Figure 3**). Using data from the Atherosclerosis Risk in Communities Study (ARIC) from the discovery phase, we performed additional conditional analyses by including the index SNPs as covariates. Conditional analysis adjusting for the arteriolar caliber index SNP rs2194025 removed the association at this locus on chromosome 5. The conditional analysis attenuated the signals of the SNPs close to the index SNP, but appeared to strengthen the associations 200 kb upstream near *TMEM161B* (**Figure 4**). This suggests the possibility of more than one functional variant affecting retinal arteriolar caliber on chromosome 5. Similar results were observed when adjusting for the venular index SNP rs17421627 [23]. The association signals were attenuated around the retinal arteriolar index SNP. However, association signals nearer *TMEM161B* and *MEF2C* were largely unaffected and instead, appeared to be strengthened (**Figure 4**).

### Associations with Clinical End-points

We also performed *in-silico* look-ups of the three index SNPs, rs2194025, rs3704461 and rs2281827, in three independent cohorts that had information on clinical macrovascular endpoints. They were hypertension (Global Blood Pressure Genetics – Global BPgen) [27], coronary artery disease (Wellcome Trust Case Control Consortium – WTCCC) [28], stroke or myocardial infarction (Heart and Vascular Health Study – HVH) [29], [30], and incident coronary artery and ischemic stroke events in a diabetic cohort (Go-DARTS) [31] (**Table 4**). A signification association was reported for rs2281827, with prevalent ischemic heart disease and incident ischemic stroke in the GoDARTS cohort. The allele that decreased arteriolar caliber was associated with increased risk of ischemic heart disease, however, the same allele was also associated with reduced risk of incident ischemic stroke (hazard ratio HR = 0.56, *P*-value = 1.14×10^−4^).

**Table 4:** Association of the three index SNPs with macrovascular diseases.

SNP (chr: position)	Ref/Effective allele (+)	Cohort	Eff allele freq	OR (95% CI)	*P*-value
rs2194025 (5: 87833992)	C/G	WTCCC (CAD) 2,000 cases/3,000 controls	0.91	1.00 (0.86–1.15)	0.96
		HVH (Stroke) 501 cases/1,314 controls	0.91	0.91 (0.69–1.19)	0.50
		HVH (MI) 1,172 cases/1,314 controls	0.91	1.01 (0.83–1.24)	0.91
		Global BPGen (HTN) ∼9,000 case control pairs	0.90	0.95 (0.87–1.03)	0.21
		GoDARTS (CAD) 541 cases/2,058 controls‡	0.91	1.13 (0.84–1.53)	0.39
		GoDARTS (Incident CAD)† 195 cases	0.91	1.00 (0.70–1.42)	0.99
		GoDARTS (Incident Ischaemic Stroke) * † 129 cases	0.91	1.06 (0.69–1.64)	0.79
		**Cohort**	**Eff allele freq**	**OR (95% CI)**	***P*** **-value**
rs3744061 (17: 72244998)	A/G	WTCCC (CAD) 2,000 cases/3,000 controls	0.45	1.05 (0.97–1.15)	0.21
		HVH (Stroke) 501 cases/1,314 controls	0.43	0.96 (0.82–1.13)	0.62
		HVH (MI) 1,172 cases/1,314 controls	0.44	1.07 (0.95–1.21)	0.26
		Global BPGen (HTN) ∼9,000 case control pairs	0.47	1.02 (0.97–1.07)	0.42
		GoDARTS (CAD) 541 cases/2,058 controls‡	0.41	0.92 (0.78–1.11)	0.39
		GoDARTS (Incident CAD)† 195 cases	0.41	0.96 (0.83–1.29)	0.75
		GoDARTS (Incident Ischaemic Stroke) * † 129 cases	0.42	0.93 (0.71–1.23)	0.62
		**Cohort**	**Eff allele freq**	**Beta (SE)**	***P*** **-value**
rs2281827 (13: 27899721)	T/C	WTCCC (CAD) 2,000 cases/3,000 controls	0.77	1.07 (0.97–1.19)	0.18
		HVH (Stroke) 501 cases/1,314 controls	0.75	0.96 (0.78–1.18)	0.70
		HVH (MI) 1,172 cases/1,314 controls	0.75	0.99 (0.85–1.15)	0.87
		Global BPGen (HTN) ∼9,000 case control pairs	0.78	1.01 (0.95–1.07)	0.86
		GoDARTS (CAD) 541 cases/2,058 controls‡	0.77	1.23 (1.01–1.50)	0.04
		GoDARTS (Incident CAD)† 195 cases	0.77	1.22 (0.94–1.59)	0.14
		GoDARTS (Incident Ischaemic Stroke) * † 129 cases	0.77	0.56 (0.42–0.75)	1.14×10^−4^

SE: standard error, OR: odds ratio, N: number of incident events, HR: hazards ratio, WTCCC: Wellcome Trust Case Control Consortium, CAD: Coronary Artery Disease, HVH: Heart and Vascular Health Study, MI: Myocardial infarction, Global BPGen: Global Blood Pressure Genetics Consortium; HTN: hypertension, GoDARTS: Genetics of Diabetes Audit and Reseach Tayside Scotland.

The allele that decreases retinal arteriolar caliber is presented as the effective allele (refer to Table 2).

* excludes cardioembolic stroke.

‡ adjusted for age, gender, BMI, history of smoking and hypertension medication.

† adjusted for age, gender, BMI, history of smoking, previous CAD events and previous ischaemic stroke events.

## Discussion

We identified one new locus on chromosome 5 which harbor variants convincingly associated with retinal arteriolar caliber in the meta-analysis of five cohorts of European ancestry. These findings were replicated in two additional independent cohorts also of European ancestry.

The locus associated with retinal arteriolar caliber on chromosome 5 spanned about 80 kb between *TMEM161B* and *MEF2C*. The nearest gene, *MEF2C*, is a regulator of cardiac morphogenesis and essential in the development of the right ventricle in mice [35], [36], [37]. Recently, *MEF2C* has also been implicated in the pathogenesis of insulin resistance, an important risk factor for vascular disease. Myostatin is thought to be a negative regulator of skeletal muscle growth. Deletion of the myostatin gene reduces insulin resistance [38] and alters skeletal muscle fiber composition. The latter is thought to be mediated by altered expression of *MEF2C* [39]. Given its known effect in regulating growth and differentiation of muscle, and recent data showing that variants at the same locus of *MEF2C* were also associated with bone mineral density, it is possible that these variants act through an effect on connective tissues that affect blood vessel morphology in general [40]. Interestingly, the locus we found on chromosome 5 near *MEFC2* in the present analysis was also found to be associated with retinal venular caliber [23]. Xing et al reported seven linkage regions each for retinal arteriolar and venular caliber, and although none of them overlapped with the findings in the current genome-wide association analysis, three of the linkage regions were common to both retinal arteriolar and venular calibers [19]. The associations of *MEF2C* locus with both retinal arteriolar and venular calibers suggest that this locus influences retinal vessel structure. Conditional analyses suggest the possibility of more than one functional mechanism at this locus, either affecting one or both of the retinal calibers.

The two loci on chromosome 13 and 17 showed less conclusive evidences of association with retinal arteriolar caliber (**Table 2** **,** **Table 3** and **Table S2**). For index SNP rs2281827 on chromosome 13, the *P*-value in discovery was 3.55×10^−7^ and it failed to replicate in the replication cohorts. Although the index SNP rs3744061 associated with retinal arteriolar caliber did show replication, this was an imputed SNP and when we looked at the next best genotyped SNP rs9916811, the combined *P*-value from discovery and replication cohorts was 1.77×10^−5^. The index SNP rs3744061 on chromosome 17 was located near a cluster of genes, *SFRS2*, *MFSD11*, *JMJD6* and *MXRA7* while the index SNP rs2281827 on chromosome 13 was found on the vascular endothelial growth factor receptor 1 (*FLT-1/VEGFR-1*). *JMJD6* is a RNA splicing regulator and recently, reduction of *JMJD6* expression has been found to alter splicing of the vascular endothelial growth factor receptor 1 (*FLT-1/VEGFR-1*), increasing levels of soluble *FLT-1* binding to the vascular endothelial growth factor (*VEGF*) and thereby inhibits angiogenesis [41]. The *VEGF* receptor locus is likely to be implicated in both microvascular and macrovascular diseases although the observed reverse association with ischemic stroke compared to coronary heart disease in the diabetics is not immediately explainable. It might be due to increased VEGF production in diabetic-related haemodynamic changes [42], larger retinal arteriolar caliber in subjects with diabetes [9], [43], [44] and possible selection bias in the diabetic individuals. More evidence from studies working on diabetics will be important to elucidate these findings.

Retinal arteriolar caliber is a dynamic anatomic trait that is affected by many physiologic and behavioral factors such as hypertension, smoking and changes in response to the cardiac cycle whereas retinal venular caliber is thought to be, in comparison, more static. In addition, measurements of the retinal arteriolar caliber tend to be less precise because there is weaker colour contrast between the arterioles and the surrounding retina, whereas venules are more easily demarcated. Hence non-differential measurement error in the measurement of retinal arteriolar caliber could be higher than retinal venular caliber leading to bias findings towards the null and reduced effect estimates. We might expect some of the genetic determinants of retinal arteriolar caliber to require larger sample sizes for more power to detect the genetic effects, if they exist. Some evidence can be seen from the differences in the intra- and inter-class correlations of vessel measurements across graders analyzing the retinal images. Comparatively, the correlations between graders for arteriolar caliber were lower than those for retinal venular caliber in each cohort (Text S1). Therefore, it is not surprising that a larger discovery sample size was required to establish an association with retinal arteriolar caliber.

We were not able to demonstrate associations between the locus for retinal arteriolar and any macrovascular disease endpoints, including coronary artery disease, myocardial infarction, hypertension, and ischemic stroke, despite the reported relationships between retinal vascular diameters and macrovascular outcomes. We previously reported one of the variants associated with retinal venular caliber on chromosome 12q24 near the *SH2B3* gene to be associated with coronary artery disease and hypertension [23]. Reasons for the failure to demonstrate any association between rs2194025 and common phenotypes of macrovascular disease endpoints in the current analysis are unclear. One possibility is that our study was not adequately powered to detect an association. We estimated the power of our study to detect an association between rs2194025 and hypertension, given the known association between retinal arteriolar caliber and hypertension. In the Blue Mountain Eye Study, the odds ratio of 5-year incident hypertension was approximately 1.25 per standard deviation (SD = 20 µm) decrease in retinal arteriolar caliber [6], [33]. The top SNP associated with retinal arteriolar caliber (rs2194025) was associated with a 1.6 µm decrease in retinal arteriolar caliber per copy of the effective allele. We estimated the odds ratio for the association between this SNP and hypertension to be 1.02. Given the allele frequency of this SNP and the sample size for the *in-silico* look-up in 9,000 case-control pairs from the Global BPGen Consortium, our study had less than 10% power to detect an association with hypertension. Small vessel disease also likely represents only one of the multiple disease pathways in the pathogenesis of macrovascular disease outcomes and might have a more prominent role in the manifestation of disease in its early stage. Both narrowed retinal arteriolar caliber at baseline and acquired changes in retinal vessel caliber over time have important roles in the clinical relevance of macrovascular disease outcomes [7], [12], [13]. With just absolute effect sizes estimated from the SNPs in our cross-sectional study, we are less equipped to finding relevant associations with clinical outcomes. Heterogeneity in the clinical phenotypes of the studies of macrovascular disease outcomes pooled across multiple studies for each genetic consortium may have also been a factor. Other studies have observed similar difficulties linking genetic loci associated with a particular risk marker and subsequently finding relevant associations with clinical outcomes from clinical epidemiological studies. For instance, SNPs associated with glycemic traits and high density lipoproteins are not consistently associated with Type 2 diabetes [45] and coronary artery disease [46].

Our study consisted of fairly homogenous groups of individuals of European ancestry and retinal arteriolar caliber was measured using similar or slightly modified photography techniques, digitalization methods and computational formulas across the study cohorts. Further work is needed to fine map existing loci to localize the causal variants and to investigate the interplay of genetic and environment modifiers in determining the retinal vessel calibers. In conclusion, we identified and confirmed a novel locus associated with retinal arteriolar caliber. These findings may shed light on the genetic influence underlying the development of microvascular disease.

## Materials and Methods

### Ethics Statement

All cohorts secured approval from their respective institutional review boards and participants gave written informed consent in accordance with the Declaration of Helsinki.

### Discovery Cohorts

The CHARGE consortium consists of large prospective studies from the United States and Europe that have genome-wide scans and well-phenotyped data, measured in a similar way across the different studies [32]. All participating studies approved guidelines for collaboration, and working groups arrived at consensus on phenotype harmonization, covariate selection and analytical plans for both individual cohort analyses and combined meta-analyses [32]. Detailed descriptions of each cohort, retinal vessels measurements and genotyping information are presented in the Text S1. Briefly, the Age Gene/Environment Susceptibility – Reykjavik Study (AGES) consists of 5,764 survivors examined between 2002 to 2006 who were original participants of Iceland’s Reykjavik Study, a random sample of 30,795 men and women living in Reykjavik in 1967 and born between 1907 and 1935 [47]. The Atherosclerosis Risk in Communities Study (ARIC) study is a prospective population-based cohort in the United States, consisting of 15,792 individuals aged 45 to 64 at baseline (1987–1989), to study the risk factors of cardiovascular diseases with yearly follow-up on clinical outcomes [48]. The Cardiovascular Heart Study (CHS) cohort is a population-based cohort study of cardiovascular risk factors in 5,201 adults of European descent recruited in 1989–1990 and 687 African-Americans enrolled in 1992 to 1993 in the United States [49]. The Rotterdam Study (RS) enrolled 7,983 residents from Rotterdam, The Netherlands who were aged 55 years and older to study neurogeriatric, cardiovascular, bone, and eye diseases and health in the elderly, with baseline examination between 1990 and 1993 [50]. The Blue Mountain Eye Study (BMES) cohort is a population-based cohort of a predominantly white population aged 49 years or older at baseline in west Sydney, Australia [24]. From the original cohort, 2,335 participants returned for follow-up examinations during 1997–1999 (BMES IIA) and 1,174 new eligible individuals participating in an Extension Study of the BMES (BMES IIB) during 1999 to 2000 formed the population for this study (BMES cross-sectional II). Only white individuals of European ancestry from the various cohorts were included in this study.

### Replication Cohorts

The Australian Twin Eye Study comprises of 1,769 twins from the Twins Eye Study in Tasmania (TEST) [26] or the Brisbane Adolescent Twins Study (BATS) with complete phenotype and genetic data. The Multi-Ethnic Study of Atherosclerosis (MESA) was initiated to look into the pathophysiology of subclinical disease development and progression and its role in clinical cardiovascular disease in 6,814 individuals aged 45 to 84 years from four ethnic groups in the United States, including African Americans, Asian, Hispanic and Whites [25]. Only the non-Hispanic white participants were included in this study.

### Retinal Vascular Caliber Measurements

Retinal vascular caliber was measured using standardized protocols and software first developed at the University of Wisconsin. They were first implemented in the ARIC [51] and CHS [52] studies, and later, with slight modifications, in the RS [1] and AGES [11]. Participants underwent film or digital retinal photography and optic disc-centered images (Early Treatment Diabetic Retinopathy Study ETDRS Field 1) were used to measure vascular caliber. In addition, pharmacological mydriasis was used in the AGES and Rotterdam studies. For ARIC, CHS and RS, the photographs of one eye were digitized using a high-resolution scanner while for AGES and BMES, digital photographs of both eyes were captured. All digital retinal images were analyzed with a semi-automated retinal vessels measurement system and the calibers of all retinal arterioles and venules were measured in an area between half and one disc-diameter from the optic disc margin. The Parr-Hubbard-Knudtson formulas were used to compute summary measures for retinal arteriolar in the six largest arterioles and venular calibers in micrometers (µm) [53]. We refer to them as the central retinal arteriolar and venular equivalents respectively.

### Genotyping

The discovery cohorts were genotyped on different genotyping platforms: Illumina HumanCNV370 for AGES and CHS, Affymetrix GeneChip SNP Array 6.0for ARIC, Illumina Infinium II HumanHap 550v3 for RS and Illumina Human670Quadv1 custom chip for BMES. All cohorts were imputed to 2.5 million on the Hapmap CEU reference panel, after extensive quality control analyses within each cohort [32].

### Statistical Analyses

Each cohort fitted an additive genetic model with one degree of freedom relating the retinal vessels to genotype dosages (0 to 2 copies) of the effective allele, adjusting for age, sex and study site whenever necessary. In addition, four multi-dimensional scaling dimensions were included for the BMES. Linear regression was used to compute regression coefficients and their standard errors (SE), using the ProbABEL program (http://mga.bionet.nsc.ru/~yurii/ABEL/) [54] in AGES, ARIC, BMES and Rotterdam and the R software (http://www.r-project.org) in CHS. Genomic control [55] was applied to each cohort before meta-analysis by multiplying the square of the genomic control inflation factor λ_gc_ to the SE to account for possible residual population structure or other confounding factors. The genomic inflation factors for the 5 cohorts ranged from 1.004 to 1.041.

### Meta-analysis

We conducted a meta-analysis combining summary results from linear regression analyses of data from five cohorts of European descent (AGES, ARIC, CHS, RS and BMES) using an inverse-variance weighted meta-analysis method with the METAL software (http://www.sph.umich.edu/csg/abecasis/Metal/index.html). Strand information was available from all the cohorts, and all results were synchronized to the forward strand. Overall meta-analysis inflation factor was 1.03. An *a priori* genome-wide significance threshold at *P*-value <5×10^−8^ was used, corresponding to a *P*-value of 0.05 with Bonferroni correction for one million independent tests.

### Replication-analyses

The two genome-wide significance SNPs from the discovery stage were further examined in two replication cohorts of European ancestry, MESA and Australian Twins Study. We also included one highly suggestive locus for which the *P*-value was less than 10^−6^. These index SNPs were examined *in-silico* in 1,769 participants from the Australian Twins Study and 2,170 whites from MESA. Retinal vascular caliber measurements in these cohorts used the same methodology as in the discovery cohorts [53], [56], [57], [58], [59], [60]. Whole-genome genotyping was done using the Illumina HumanHap610W array for the Australian Twins and the Affymetrix Genome-Wide Human SNP Array 6.0 for MESA participants.

### Conditional Analyses

Retinal arteriolar and retinal venular vessels are moderately correlated [33], [34]. The primary analyses were subsequently repeated including retinal venular caliber as a covariate. Conditional analyses were performed at chromosome 5 in two ways on ARIC data, (i) by including retinal arteriolar caliber index SNP rs2194025 as a covariate while exploring additional retinal arteriolar associated SNPs in the region and (ii) by including the index SNP associated with retinal venular caliber rs17421627 as a covariate to determine if the SNPs at the same locus remain associated with arteriolar caliber independent of their associations with venular caliber.

### Analyses with Clinical Endpoints

Finally, we performed *in-silico* look-ups of the novel and suggestive variants for several macrovascular outcomes including, coronary artery disease from the Wellcome Trust Case Control Consortium (WTCCC) [28], stroke and myocardial infarction from the Heart and Vascular Health (HVH) Study [29], [30], hypertension from the Global Blood Pressure Genetics (Global BPgen) Consortium [27] and incident coronary artery disease/ischemic stroke events from the Genetics of Diabetes Audit and Research in Tayside Scotland (GoDARTS). To examine the association of these with macrovascular outcomes, we obtained summary association statistics for the index SNPs. The WTCCC coronary artery study consisted of 2,000 coronary artery cases and 3,000 common controls [28]. The HVH study was comprised of 501 stroke cases, 1,172 myocardial infarction and 1,314 controls [29], [30]. In the Global BPGen study, there were approximately 9,000 hypertensive cases and 10,000 controls. The GoDARTS cohort consisted of 3,328 Type 2 Diabetic individuals with a mean follow up time of four years. The case-control analysis for coronary heart disease included 541 cases and 2,038 controls. Survival analysis to incident macrovascular events included 195 coronary artery events and 129 ischemic stroke events.

## Supporting Information

Table S1 **Association of index SNP with retinal arteriolar caliber at three top loci for each discovery cohort and meta-analysis additionally adjusted for hypertension and diabetes status in each discovery cohort.**
(DOC)

Table S2 **Association of next best index SNPs directly genotyped for the three loci.**
(DOC)

Table S3 **Membership of the Wellcome Trust Case Control Consortium 2 (WTCCC2).**
(DOC)

Table S4 **Membership of the Global Blood Pressure Genetics (Global BPgen) consortium.**
(DOC)

Text S1 (DOC)
