# Supplementary material for: Genetic Loci for Retinal Arteriolar Microcirculation
Source: PLoS One. 2013 Jun 12;8(6):e65804. doi: 10.1371/journal.pone.0065804 (PMC3680438; doi:10.1371/journal.pone.0065804)
Supplement: Table S1 — Association of index SNP with retinal arteriolar caliber at three top loci for each discovery cohort and meta-analysis additionally adjusted for hypertension and diabetes status in each discovery cohort. (DOC) [file pone.0065804.s001.doc]

| SNP (chr: position) | Ref/Effective allele (+) | Cohort | Eff allele freq | Beta (SE) | *P*-value | Genes of interest |
| --- | --- | --- | --- | --- | --- | --- |
| rs2194025 (5: 87833992) | C/G | AGES | 0.95 | -1.30 (0.79) | 9.74 x 10^-2^ | *TMEM16B* |
|  |  | ARIC | 0.90 | -1.25 (0.36) | 4.33 x 10^-4^ | *MEF2C* |
|  |  | CHS | 0.91 | -3.14 (0.99) | 2.00 x 10^-3^ |  |
|  |  | RS | 0.90 | -1.46 (0.48) | 2.29 x 10^-3^ |  |
|  |  | BMES | 0.90 | -2.45 (0.74) | 9.72 x 10^-4^ |  |
|  |  | Cohort | Eff allele freq | Beta (SE) | *P*-value | Genes of interest |
| rs3744061 (17: 72244998) | A/G | AGES | 0.46 | -0.96 (0.35) | 6.23 x 10^-3^ | *SFRS2* |
|  |  | ARIC | 0.44 | -1.03 (0.21) | 1.53 x 10^-6^ | *MFSD11* |
|  |  | CHS | 0.43 | -1.16 (0.60) | 6.00 x 10^-2^ | *JMJD6* |
|  |  | RS | 0.42 | -0.27 (0.30) | 3.72 x 10^-1^ | *MXRA7* |
|  |  | BMES | 0.45 | -0.52 (0.45) | 2.43 x 10^-1^ |  |
|  |  | Cohort | Eff allele freq | Beta (SE) | *P*-value | Genes of interest |
| rs2281827 (13: 27899721) | T/C | AGES | 0.78 | -1.27 (0.44) | 4.09 x 10^-3^ | *FLT1* |
|  |  | ARIC | 0.77 | -0.40 (0.26) | 1.18 x 10^-1^ |  |
|  |  | CHS | 0.73 | -1.08 (0.70) | 1.20 x 10^-1^ |  |
|  |  | RS | 0.78 | -1.61 (0.37) | 1.47 x 10^-5^ |  |
|  |  | BMES | 0.76 | -0.96 (0.52) | 6.46 x 10^-2^ |  |

SE: standard error, AGES: Age Gene/Environment Susceptibility – Reykjavik Study, ARIC: Atherosclerosis Risk in Communities Study, CHS: Cardiovascular Health Study, RS: Rotterdam Study, BMES: Blue Mountains Eye Study.

The allele that decreases retinal arteriolar caliber is presented as the effective allele.

Diabetes mellitus, in AGES, ARIC, CHS and BMES, is defined by self-reported, fasting blood glucose ≥ 126 mg/dL (7.0 mmol/L), and/or medication use. In RS, diabetes mellitus was defined as defined as a non-fasting glucose level ≥ 11.0 mmol/l and/or antidiabetic medication use.
