# Supplementary material for: Genetic Loci for Retinal Arteriolar Microcirculation
Source: PLoS One. 2013 Jun 12;8(6):e65804. doi: 10.1371/journal.pone.0065804 (PMC3680438; doi:10.1371/journal.pone.0065804)
Supplement: Table S2 — Association of next best index SNPs directly genotyped for the three loci. (DOC) [file pone.0065804.s002.doc]

| SNP (chr: position) | Next best SNP (r^2^) (G/I) | Ref/Effective allele (+) | Cohort | Eff allele freq | Beta (SE) | *P*-value | Genes of interest |
| --- | --- | --- | --- | --- | --- | --- | --- |
| rs2194025 (5: 87833992) | rs2194026 (r^2^ = 1) (GIGGG) | G/T | Discovery cohorts combined | 0.91 | -1.56 (0.25) | 2.35 x 10 ^-10^ | *TMEM16B* |
|  |  |  | MESA Whites | 0.91 | -2.01 (0.76) | 8.00 x 10^-3^ | *MEF2C* |
|  |  |  | Australian Twins | 0.91 | -1.14 (0.83) | 1.68 x 10^-1^ |  |
|  |  |  | Replication cohorts combined | 0.91 | -1.62 (0.56) | 3.97 x 10^-3^ |  |
|  |  |  | Discovery + Replication cohorts combined | 0.90 | -1.56 (0.22) | 3.43 x 10^-12^ |  |
|  |  |  | Cohort | Eff allele freq | Beta (SE) | *P*-value | Genes of interest |
| rs3744061 (17: 72244998) | rs9916811 (r^2^ = 0.844) (GGGGG) | C/T | Discovery cohorts combined | 0.48 | -0.75 (0.14) | 1.05 x 10^-8^ | *SFRS2* |
|  |  |  | MESA Whites | 0.48 | -0.49 (0.51) | 2.50 x 10^-1^ | *MFSD11* |
|  |  |  | Australian Twins | 0.50 | 1.70 (0.49) | 4.84 x 10^-4^ | *JMJD6* |
|  |  |  | Replication cohorts combined | 0.49 | 0.44 (0.32) | 1.62 x 10^-1^ | *MXRA7* |
|  |  |  | Discovery + Replication cohorts combined | 0.48 | -0.55 (0.13) | 1.77 x 10^-5^ |  |
|  |  |  | Cohort | Eff allele freq | Beta (SE) | *P*-value | Genes of interest |
| rs2281827 (13: 27899721) | rs722503 (r^2^ = 0.529) (GIGGG) | T/C | Discovery cohorts combined | 0.76 | -0.84 (0.17) | 9.38 x 10^-7^ | *FLT1* |
|  |  |  | MESA Whites | 0.74 | -0.25 (0.51) | 6.30 x 10^-1^ |  |
|  |  |  | Australian Twins | 0.25 | -0.57 (0.57) | 3.08 x 10^-1^ |  |
|  |  |  | Replication cohorts combined | 0.52 | -0.40 (0.38) | 2.94 x 10^-1^ |  |
|  |  |  | Discovery + Replication cohorts combined | 0.72 | -0.72 (0.16) | 9.75 x 10^-7^ |  |

SE: standard error, OR: odds ratio, MESA: Multi-Ethnic Study of Atherosclerosis.

The allele that decreases retinal arteriolar caliber is presented as the effective allele (refer to Table 2).

r^2^: linkage disequilibrium r^2^ between the SNP reported in the main paper and the next best index SNP from SNAP (<http://www.broadinstitute.org/mpg/snap/ldsearch.php>) using Europeans from Hapmap II.

(G/I): Indicates if the SNP is directly genotyped (G) or imputed (I), given in the following order: AGES: Age Gene/Environment Susceptibility – Reykjavik Study, ARIC: Atherosclerosis Risk in Communities Study, CHS: Cardiovascular Health Study, RS: Rotterdam Study, BMES: Blue Mountains Eye Study.
