# Supplementary material for: Genetic Loci for Retinal Arteriolar Microcirculation
Source: PLoS One. 2013 Jun 12;8(6):e65804. doi: 10.1371/journal.pone.0065804 (PMC3680438; doi:10.1371/journal.pone.0065804)
Supplement: Table S3 — Membership of the Wellcome Trust Case Control Consortium 2 (WTCCC2). (DOC) [file pone.0065804.s003.doc]

**Table S3 – Membership of Wellcome Trust Case Control Consortium 2**

Management Committee

Peter Donnelly (Chair)^1,2^, Ines Barroso (Deputy Chair)^3^, Jenefer M Blackwell^4, 5^, Elvira Bramon^6^ , Matthew A Brown^7^ , Juan P Casas^8^ , Aiden Corvin^9^, Panos Deloukas^3^, Audrey Duncanson^10^, Janusz Jankowski^11^, Hugh S Markus^12^, Christopher G Mathew^13^, Colin NA Palmer^14^, Robert Plomin^15^, Anna Rautanen^1^, Stephen J Sawcer^16^, Richard C Trembath^13^, Ananth C Viswanathan^17^, Nicholas W Wood^18^

Data and Analysis Group

Chris C A Spencer^1^, Gavin Band^1^, Céline Bellenguez^1^, Colin Freeman^1^, Garrett Hellenthal^1^, Eleni Giannoulatou^1^, Matti Pirinen^1^, Richard Pearson^1^, Amy Strange^1^, Zhan Su^1^, Damjan Vukcevic^1^, Peter Donnelly^1,2^

DNA, Genotyping, Data QC and Informatics Group

Cordelia Langford^3^, Sarah E Hunt^3^, Sarah Edkins^3^, Rhian Gwilliam^3^, Hannah Blackburn^3^, Suzannah J Bumpstead^3^, Serge Dronov^3^, Matthew Gillman^3^, Emma Gray^3^, Naomi Hammond^3^, Alagurevathi Jayakumar^3^, Owen T McCann^3^, Jennifer Liddle^3^, Simon C Potter^3^, Radhi Ravindrarajah^3^, Michelle Ricketts^3^, Matthew Waller^3^, Paul Weston^3^, Sara Widaa^3^, Pamela Whittaker^3^, Ines Barroso^3^, Panos Deloukas^3^**.**

Publications Committee

Christopher G Mathew (Chair)^13^, Jenefer M Blackwell^4,5^, Matthew A Brown^7^, Aiden Corvin^9^, Mark I McCarthy^19^, Chris C A Spencer^1^

^1^ Wellcome Trust Centre for Human Genetics, Roosevelt Drive, Oxford OX3 7LJ, UK;

^2^ Dept Statistics, University of Oxford, Oxford OX1 3TG, UK;

^3^ Wellcome Trust Sanger Institute, Wellcome Trust Genome Campus, Hinxton, Cambridge CB10 1SA, UK;

^4^ Telethon Institute for Child Health Research, Centre for Child Health Research, University of Western Australia, 100 Roberts Road, Subiaco, Western Australia 6008;

^5^ Cambridge Institute for Medical Research, University of Cambridge School of Clinical Medicine, Cambridge CB2 0XY, UK;

^6^ Department of Psychosis Studies, NIHR Biomedical Research Centre for Mental Health at the Institute of Psychiatry, King’s College London and The South London and Maudsley NHS Foundation Trust, Denmark Hill, London SE5 8AF, UK;

^7^ Diamantina Institute of Cancer, Immunology and Metabolic Medicine, Princess Alexandra Hospital, University of Queensland, Brisbane, Queensland, Australia;

^8^ Dept Epidemiology and Population Health, London School of Hygiene and Tropical Medicine, London WC1E 7HT and Dept Epidemiology and Public Health, University College London WC1E 6BT, UK;

^9^ Neuropsychiatric Genetics Research Group, Institute of Molecular Medicine, Trinity College Dublin, Dublin 2, Eire;

^10^ Molecular and Physiological Sciences, The Wellcome Trust, London NW1 2BE;

^11^ Centre for Digestive Diseases, Queen Mary University of London, London E1 2AD, UK and Digestive Diseases Centre, Leicester Royal Infirmary, Leicester LE7 7HH, UK and Department of Clinical Pharmacology, Old Road Campus, University of Oxford, Oxford OX3 7DQ, UK;

^12^ Clinical Neurosciences, St George's University of London, London SW17 0RE;

^13^ King’s College London Dept Medical and Molecular Genetics, School of Medicine, Guy’s Hospital, London SE1 9RT, UK;

^14^ Biomedical Research Centre, Ninewells Hospital and Medical School, Dundee DD1 9SY, UK;

^15^ King’s College London Social, Genetic and Developmental Psychiatry Centre, Institute of Psychiatry, Denmark Hill, London SE5 8AF, UK;

^16^ University of Cambridge Dept Clinical Neurosciences, Addenbrooke’s Hospital, Cambridge CB2 0QQ, UK;

^17^ NIHR Biomedical Research Centre for Ophthalmology, Moorfields Eye Hospital NHS Foundation Trust and UCL Institute of Ophthalmology, London EC1V 2PD, UK;

^18^ Dept Molecular Neuroscience, Institute of Neurology, Queen Square, London WC1N 3BG, UK;

^19^ Oxford Centre for Diabetes, Endocrinology and Metabolism (ICDEM), Churchill Hospital, Oxford OX3 7LJ, UK.
