# Supplementary material for: Genetic Loci for Retinal Arteriolar Microcirculation
Source: PLoS One. 2013 Jun 12;8(6):e65804. doi: 10.1371/journal.pone.0065804 (PMC3680438; doi:10.1371/journal.pone.0065804)
Supplement: Table S4 — Membership of the Global Blood Pressure Genetics (Global BPgen) consortium. (DOC) [file pone.0065804.s004.doc]

**Table S4 – Membership of the Global Blood Pressure Genetics (Global BPgen) consortium**

Christopher Newton-Cheh^1,2,3^, Toby Johnson^4,5,6^, Vesela Gateva^7^, Martin D Tobin^8^, Murielle Bochud^5^, Lachlan Coin^9^, Samer S Najjar^10^, Jing Hua Zhao^11,12^, Simon C Heath^13^, Susana Eyheramendy^14,15^, Konstantinos Papadakis^16^, Benjamin F Voight^1,3^, Laura J Scott^7^, Feng Zhang^17^, Martin Farrall^18,19^, Toshiko Tanaka^20,21^, Chris Wallace^22,23^, John C Chambers^9^, Kay-Tee Khaw^12,24^, Peter Nilsson^25^, Pim van der Harst^26^, Silvia Polidoro^27^, Diederick E Grobbee^28^, N Charlotte Onland-Moret^28,29^, Michiel L Bots^28^, Louise V Wain^8^, Katherine S Elliott^19^, Alexander Teumer^30^, Jian’an Luan^11^, Gavin Lucas^31^, Johanna Kuusisto^32^, Paul R Burton^8^, David Hadley^16^, Wendy L McArdle^33^, Wellcome Trust Case Control Consortium^34^, Morris Brown^35^, Anna Dominiczak^36^, Stephen J Newhouse^22^, Nilesh J Samani^37^, John Webster^38^, Eleftheria Zeggini^19,39^, Jacques S Beckmann^4,40^, Sven Bergmann^4,6^, Noha Lim^41^, Kijoung Song^41^, Peter Vollenweider^42^, Gerard Waeber^42^, Dawn M Waterworth^41^, Xin Yuan^41^, Leif Groop^43,44^, Marju Orho-Melander^25^, Alessandra Allione^27^, Alessandra Di Gregorio^27,45^, Simonetta Guarrera^27^, Salvatore Panico^46^, Fulvio Ricceri^27^, Valeria Romanazzi^27,45^, Carlotta Sacerdote^47^, Paolo Vineis^9,27^, Ineˆs Barroso^12,39^, Manjinder S Sandhu^11,12,24^, Robert N Luben^12,24^, Gabriel J. Crawford^3^, Pekka Jousilahti^48^, Markus Perola^48,49^, Michael Boehnke^7^, Lori L Bonnycastle^50^, Francis S Collins^50^, Anne U Jackson^7^, Karen L Mohlke^51^, Heather M Stringham^7^, Timo T Valle^52^, Cristen J Willer^7^, Richard N Bergman^53^, Mario A Morken^50^, Angela Do¨ring^15^, Christian Gieger^15^, Thomas Illig^15^, Thomas Meitinger^54,55^, Elin Org^56^, Arne Pfeufer^54^, H Erich Wichmann^15,57^, Sekar Kathiresan^1,2,3^, Jaume Marrugat^31^, Christopher J O’Donnell^58,59^, Stephen M Schwartz^60,61^, David S Siscovick^60,61^, Isaac Subirana^31,62^, Nelson B Freimer^63^, Anna-Liisa Hartikainen^64^, Mark I McCarthy^19,65,66^, Paul F O’Reilly^9^, Leena Peltonen^39,49^, Anneli Pouta^64,67^, Paul E de Jong^68^, Harold Snieder^69^, Wiek H van Gilst^26^, Robert Clarke^70^, Anuj Goel^18,19^, Anders Hamsten^71^, John F Peden^18,19^, Udo Seedorf^72^, Ann-Christine Syva¨nen^73^, Giovanni Tognoni^74^, Edward G Lakatta^10^, Serena Sanna^75^, Paul Scheet^76^, David Schlessinger^77^, Angelo Scuteri^78^, Marcus Do¨rr^79^, Florian Ernst^30^, Stephan B Felix^79^, Georg Homuth^30^, Roberto Lorbeer^80^, Thorsten Reffelmann^79^, Rainer Rettig^81^, Uwe Vo¨lker^30^, Pilar Galan^82^, Ivo G Gut^13^, Serge Hercberg^82^, G Mark Lathrop^13^, Diana Zeleneka^13^, Panos Deloukas^12,39^, Nicole Soranzo^17,39^,

Frances M Williams^17^, Guangju Zhai^17^, Veikko Salomaa^48^, Markku Laakso^32^, Roberto Elosua^31,62^, Nita G Forouhi^11^, Henry Vo¨lzke^80^, Cuno S Uiterwaal^28^, Yvonne T van der Schouw^28^, Mattijs E Numans^28^, Giuseppe Matullo^27,45^, Gerjan Navis^68^, Go¨ran Berglund^25^, Sheila A Bingham^12,83^, Jaspal S Kooner^84^, John M Connell^36^, Stefania Bandinelli^85^, Luigi Ferrucci^21^, Hugh Watkins^18,19^, Tim D Spector^17^, Jaakko Tuomilehto^52,86,87^, David Altshuler^1,3,88,89^, David P Strachan^16^, Maris Laan^56^, Pierre Meneton^90^, Nicholas J Wareham^11,12^, Manuela Uda^75^, Marjo-Riitta Jarvelin^9,67,91^, Vincent Mooser^41^, Olle Melander^25^, Ruth JF Loos^11,12^, Paul Elliott^9^, Goncalo R Abecasis^92^, Mark Caulfield^22^, Patricia B Munroe^22^

1. Center for Human Genetic Research, Massachusetts General Hospital, 185 Cambridge Street, Boston, MA 02114, USA

2. Cardiovascular Research Center, Massachusetts General Hospital, Boston, Massachusetts 02114, USA

3. Program in Medical and Population Genetics, Broad Institute of Harvard and Massachusetts Institute of Technology, Cambridge, Massachusetts, 02142, USA

4. Department of Medical Genetics, University of Lausanne, 1005 Lausanne, Switzerland

5. University Institute for Social and Preventative Medicine, Centre Hospitalier Universitaire Vaudois (CHUV) and University of Lausanne, 1005 Lausanne, Switzerland

6. Swiss Institute of Bioinformatics, Switzerland

7. Department of Biostatistics and Center for Statistical Genetics, University of Michigan, Ann Arbor, MI 48109, USA

8. Departments of Health Sciences & Genetics, Adrian Building, University of Leicester, University Road, Leicester LE1 7RH

9. Department of Epidemiology and Public Health, Imperial College London, St Mary’s Campus, Norfolk Place, London W2 1PG, UK

10. Laboratory of Cardiovascular Science, Intramural Research Program, National Institute on Aging, National Institutes of Health,

Baltimore, Maryland, USA 21224

11. MRC Epidemiology Unit, Institute of Metabolic Science, Addenbrooke’s Hospital, Cambridge CB2 0QQ, UK

12. Cambridge - Genetics of Energy Metabolism (GEM) Consortium, Cambridge, UK

13. Centre National de Génotypage, 2 rue Gaston Crémieux, CP 5721, 91 057 Evry Cedex, France

14. Pontificia Universidad Catolica de Chile, Vicuña Mackenna 4860, Facultad de Matematicas, Casilla 306, Santiago 22, Chile, 7820436

15. Institute of Epidemiology, Helmholtz Zentrum München, German Research Centre for Environmental Health, 85764 Neuherberg, Germany

16. Division of Community Health Sciences, St George’s, University of London, London SW17 0RE, UK

17. Department of Twin Research & Genetic Epidemiology, King’s College London, London SE1 7EH

18. Department of Cardiovascular Medicine, University of Oxford

19. The Wellcome Trust Centre for Human Genetics, Roosevelt Drive, Oxford, OX3 7BN, UK

20. Medstar Research Institute, 3001 S. Hanover Street, Baltimore, MD 21250, USA

21. Clinical Research Branch, National Institute on Aging, Baltimore, MD, 21250 USA

22. Clinical Pharmacology and The Genome Centre, William Harvey Research Institute, Barts and The London School of Medicine and Dentistry, Queen Mary University of London, London EC1M 6BQ

23. JDRF/WT Diabetes and Inflammation Laboratory, Cambridge Institute for Medical Research University of Cambridge, Wellcome Trust/MRC Building, Addenbrooke’s Hospital Cambridge, CB2 0XY

24. Department of Public Health and Primary Care, Institute of Public Health, University of Cambridge, Cambridge CB2 2SR, UK

25. Department of Clinical Sciences, Lund University, Malmo University Hospital, SE-20502 Malmo, Sweden26. Department of Cardiology University Medical Center Groningen, University of Groningen, Hanzeplein 1, 9700 RB Groningen, The Netherlands

27. ISI Foundation (Institute for Scientific Interchange), Villa Gualino, Torino, 10133, Italy

28. Julius Center for Health Sciences and Primary Care, University Medical Center Utrecht, STR 6.131, PO Box 85500, 3508 GA Utrecht, The Netherlands

29. Complex Genetics Section, Department of Medical Genetics - DBG, University Medical Center Utrecht, STR 2.2112, PO Box 85500, 3508 GA Utrecht, The Netherlands.

30. Interfaculty Institute for Genetics and Functional Genomics, Ernst-Moritz-Arndt-University Greifswald, 17487 Greifswald, Germany

31. Cardiovascular Epidemiology and Genetics, Institut Municipal d’Investigació Mèdica, Barcelona, Spain

32. Department of Medicine University of Kuopio 70210 Kuopio, Finland

33. ALSPAC Laboratory, Department of Social Medicine, University of Bristol, BS8 2BN, UK

34. A full list of authors is provided in the supplementary methods online.

35. Clinical Pharmacology Unit, University of Cambridge, Addenbrookes Hospital, Cambridge, UK CB2 2QQ

36. BHF Glasgow Cardiovascular Research Centre, University of Glasgow, Glasgow, UK G12 8TA

37. Department of Cardiovascular Science, University of Leicester, Glenfield Hospital, Groby Road, Leicester, LE3 9QP, UK

38. Aberdeen Royal Infirmary, Aberdeen, UK

39. Wellcome Trust Sanger Institute, Wellcome Trust Genome Campus, Hinxton, Cambridge CB10 1SA, UK

40. Service of Medical Genetics, Centre Hospitalier Universitaire Vaudois (CHUV), Lausanne, 1011, Switzerland

41. Genetics Division, GlaxoSmithKline, King of Prussia, PA 19406, USA

42. Department of Internal Medicine, Centre Hospitalier Universitaire Vaudois (CHUV) 1011 Lausanne, Switzerland

43. Department of Clinical Sciences, Diabetes and Endocrinology Research Unit, University Hospital, Malmö

44. Lund University, Malmö S-205 02, Sweden

45. Department of Genetics, Biology and Biochemistry, University of Torino, Torino, 10126, Italy

46. Department of Clinical and Experimental Medicine, Federico II University, Naples, 80100, Italy

47. Unit of Cancer Epidemiology, University of Turin and Centre for Cancer Epidemiology and Prevention (CPO Piemonte), Turin, 10126, Italy

48. National Institute for Welfare and Health P.O. Box 30, FI-00271 Helsinki, Finland

49. Institute for Molecular Medicine Finland FIMM, University of Helsinki and National Public Health Institute

50. Genome Technology Branch, National Human Genome Research Institute, Bethesda, MD 20892, USA

51. Department of Genetics, University of North Carolina, Chapel Hill, NC 27599, USA

52. Diabetes Unit, Department of Epidemiology and Health Promotion, National Public Health Institute, 00300 Helsinki, Finland

53. Physiology and Biophysics USC School of Medicine 1333 San Pablo Street, MMR 626 Los Angeles, California 90033

54. Institute of Human Genetics, Helmholtz Zentrum München, German Research Centre for Environmental Health, 85764 Neuherberg, Germany

55. Institute of Human Genetics, Technische Universität München, 81675 Munich, Germany

56. Institute of Molecular and Cell Biology, University of Tartu, 51010 Tartu, Estonia

57. Ludwig Maximilians University, IBE, Chair of Epidemiology, Munich

58. Cardiovascular Research Center and Cardiology Division, Massachusetts General Hospital, Boston, Massachusetts 02114, USA

59. Framingham Heart Study and National, Heart, Lung, and Blood Institute, Framingham, Massachusetts 01702, USA

60. Cardiovascular Health Research Unit, Departments of Medicine and Epidemiology, University of Washington, Seattle, Washington, 98101 USA

61. Department of Epidemiology, University of Washington, Seattle, Washington, 98195 USA

62. CIBER Epidemiología y Salud Pública, Barcelona, Spain

63. Center for Neurobehavioral Genetics, Gonda Center, Room 3506, 695 Charles E Young Drive South, Box 951761, UCLA, Los Angeles, CA 90095.

64. Department of Clinical Sciences/Obstetrics and Gynecology, P.O. Box 5000 Fin-90014, University of Oulu, Finland

65. Oxford Centre for Diabetes, Endocrinology and Metabolism, University of Oxford, Churchill Hospital, Old Road, Headington, Oxford OX3 7LJ, UK

66. Oxford NIHR Biomedical Research Centre, Churchill Hospital, Old Road, Headington, Oxford, UK OX3 7LJ

67. Department of Child and Adolescent Health, National Public Health Institute (KTL), Aapistie 1, P.O. Box 310, FIN-90101 Oulu, Finland

68. Division of Nephrology, Department of Medicine University Medical Center Groningen, University of Groningen, Hanzeplein 1,

9700 RB Groningen, The Netherlands

69. Unit of Genetic Epidemiology and Bioinformatics, Department of Epidemiology University Medical Center Groningen, University of Groningen, Hanzeplein 1, 9700 RB Groningen, The Netherlands

70. Clinical Trial Service Unit and Epidemiological Studies Unit (CTSU), University of Oxford, Richard Doll Building, Roosevelt Drive, Oxford, OX3 7LF, UK

71. Atherosclerosis Research Unit, Department of Medicine Solna, Karolinska Institutet, Karolinska University Hospital Solna, Building L8:03, S-17176 Stockholm, Sweden

72. Leibniz-Institut fü r Arterioskleroseforschung an der Universität Münster, Domagkstr. 3, D-48149, Münster, Germany

73. Molecular Medicine, Department of Medical Sciences, Uppsala University, SE-751 85 Uppsala, Sweden

74. Consorzio Mario Negri Sud, Via Nazionale, 66030 Santa Maria Imbaro (Chieti), Italy

75. Istituto di Neurogenetica e Neurofarmacologia, CNR, Monserrato, 09042 Cagliari, Italy

76. Department of Epidemiology, Univ. of Texas M. D. Anderson Cancer Center, Houston, TX 77030

77. Laboratory of Genetics, Intramural Research Program, National Institute on Aging, National Institutes of Health, Baltimore, Maryland, USA 21224

78. Unitá Operativa Geriatria, Istituto Nazionale Ricovero e Cura per Anziani (INRCA) IRCCS, Rome, Italy

79. Department of Internal Medicine B, Ernst-Moritz-Arndt-University Greifswald, 17487 Greifswald, Germany

80. Institute for Community Medicine, Ernst-Moritz-Arndt-University Greifswald, 17487 Greifswald, Germany

81. Institute of Physiology, Ernst-Moritz-Arndt-University Greifswald, 17487 Greifswald, Germany

82. U557 Institut National de la Santé et de la Recherche Médicale, U1125 Institut National de la Recherche Agronomique, Université Paris 13, 74 rue Marcel Cachin, 93017 Bobigny Cedex, France

83. MRC Dunn Human Nutrition Unit, Wellcome Trust/MRC Building, Cambridge CB2 0XY, U.K

84. National Heart and Lung Institute, Imperial College London SW7 2AZ

85. Geriatric Rehabilitation Unit, Azienda Sanitaria Firenze (ASF), 50125, Florence, Italy

86. Department of Public Health, University of Helsinki, 00014 Helsinki, Finland

87. South Ostrobothnia Central Hospital, 60220 Seinä joki, Finland

88. Department of Medicine and Department of Genetics, Harvard Medical School, Boston, Massachusetts 02115, USA

89. Diabetes Unit, Massachusetts General Hospital, Boston, Massachusetts 02114, USA

90. U872 Institut National de la Santé et de la Recherche Médicale, Faculté de Médecine Paris Descartes, 15 rue de l’Ecole de Médecine, 75270 Paris Cedex, France

91. Institute of Health Sciences and Biocenter Oulu, Aapistie 1, FIN-90101, University of Oulu, Finland

92. Center for Statistical Genetics, Department of Biostatistics, University of Michigan, Ann Arbor, Michigan 48109 USA
