# Supplementary material for: Genetic Loci for Retinal Arteriolar Microcirculation
Source: PLoS One. 2013 Jun 12;8(6):e65804. doi: 10.1371/journal.pone.0065804 (PMC3680438; doi:10.1371/journal.pone.0065804)
Supplement: Text S1 — (DOC) [file pone.0065804.s005.doc]

**Genetic Loci for Retinal Arteriolar Microcirculation**

**Text S1**

Text S1 contains the following information:

1. Sample selection and retinal vascular caliber measurements for each study cohort.
   1. Discovery cohorts
      1. Age, Gene/Environment Susceptibility – Reykjavik Study (AGES)
      2. Atherosclerosis Risk in Communities Study (ARIC)
      3. Cardiovascular Heart Study (CHS)
      4. Rotterdam Study (RS)
      5. Blue Mountain Eye Study (BMES)
   2. Replication cohorts
      1. Australian Twin Study
      2. Multi-Ethnic Study of Atherosclerosis (MESA)
2. Genotyping quality control filters and imputation of discovery cohorts
3. Meta-analysis
4. Analyses with Macrovascular diseases
5. References
6. **Sample selection and retinal vascular caliber measurements for each study cohort.**
   1. Discovery cohorts
      1. *Age Gene/Environment Susceptibility – Reykjavik Study (AGES)*

The original Reykjavik Study cohort consisted of a random sample of 30,795 men and women born from 1907 to 1935 and residing in Reykjavik in 1967 [1]. The AGES study re-examined 5,764 survivors from the original study. Among these, 3,219 participants were genotyped on the Illumina 370CNV BeadChip array. Sample exclusion criteria included sample failure, genotype mismatch with reference panel and gender discordance. Finally, 2,949 participants had both genome wide data and retinal vessel measurements.

Retinal images of each eye, centered on the macula and optic disc, were captured digitally using a 6.3 megapixel Canon R6 nonmydriatic camera with a Canon D-60 camera after maximal pharmacologic pupil dilation. Images with suboptimal quality, judged by a trained photographer, were retaken immediately. Throughout the study, the Ocular Epidemiology Reading Center (Madison, Wisconsin) provided routine feedback for the assessment of retinal images. Using a computer-assisted program and a standard protocol, measurements of retinal vessel caliber were made for all arterioles and venules in the area between 0.5 and 1.0 disc diameters from the optic disc margin [2]. Individual vessels measurements were combined by the Parr-Hubbard-Knudston formulas and summarized as the central retinal arteriole and venular equivalents (CRAE, CRVE) [3]. The inter-grader and intra-grader intra-class correlation coefficients ranged from 0.85 to 0.95 for CRAE and were consistently at 0.99 for CRVE.

In addition, the study was approved by the Icelandic National Bioethics Committee, VSN: 00-063 with written informed consent from all participants [4].

- - 1. *Atherosclerosis Risk in Communities Study (ARIC)*

The ARIC study is a prospective population-based study to investigate the etiology of atherosclerosis in four different US communities including non-Hispanic whites and African Americans [5]. Detailed study design can be found on the ARIC study website (<http://www.cscc.unc.edu/aric/>). At baseline examination in 1987 to 1989, participants were between the age of 45 and 64. Blood was drawn at baseline for DNA extraction. Participants were genotyped on the Affymetrix Genome-Wide Human SNP Array 6.0 at the Broad Institute. For this study, only the non-Hispanic whites are included.

Retinal photos were taken at examination 3. Briefly, a 45° nonstereoscopic color retinal photograph was taken of one eye of each participant using a fundus camera that does not require pharmacologic dilation of the pupil (Canon CR-45UAF; Canon USA, Inc., Lake Success, New York). At the central reading center, a technician digitized the color slides at 3175 dots/inch resolution using a 35-mm film scanner (Nikon LS 3510 AF, Tokyo, Japan). The measurement module was custom programmed in Khoros (public domain image processing software from the University of New Mexico - Albuquerque) and the retinal measurements were summarized in micrometer (μm) using the Parr-Hubbard-Knudtson formulas [2,3]. Among three graders, the intra-grader and inter-grader intra-class correlation coefficients were 0.69 and 0.74 for CRAE and 0.89 and 0.77 for CRVE [2].

- - 1. *Cardiovascular Health Study (CHS)*

The CHS is a population-based cohort study of risk factors for coronary heart disease and stroke in 5,201 persons, including 4,925 Europeans recruited in 1989 – 1990 [6]. An additional 687 African-Americans were enrolled into the study subsequently. At baseline, DNA was extracted from blood samples of participants who gave consent to genetic testing. The participants were genotyped on the Illumina 370CNV Duo BeadChip at the General Clinical Research Center's Phenotyping/Genotyping Laboratory at Cedars-Sinai. There were 1,263 participants, who were free of clinical cardiovascular disease at baseline and had both genotype data and retinal arteriole vessel measurements available for this study. Only those of European ancestry were included for this study.

Similar to the ARIC study, a 45° retinal photograph centered between the optic disc and the macula was obtained of one randomly selected eye using a non-mydriatic fundus camera after 5 minutes of dark adaptation (Canon CR-45UAF; Canon USA, Inc., Lake Success, New York). Photographs were digitized with a high-resolution scanner and evaluated according to a standardized protocol at the Fundus Photograph Reading Center in Madison, Wisconsin. The retinal measurements were summarized as CRAE and CRVE (in μm) using the Parr-Hubbard-Knudtson formulas [2,3]. The intra-grader and inter-grader intra-class correlation coefficients ranged from 0.67 to 0.91.

- - 1. *Rotterdam Study (RS)*

The Rotterdam Study is a population-based cohort study conducted in a district of Rotterdam to examine the determinants of neurogeriatric, cardiovascular, bone, and eye diseases and health in the elderly [7]. Baseline measurements were obtained from 1990 to 1993 and consisted of an interview at home and two visits to the research center for physical examination, with written informed consent. In addition, DNA was extracted from all participants who consented to genotyping at baseline. Genotyping was performed in high-quality extracted DNA using the Illumina Infinum II HumanHap550chip v3.0 beadchip. Finally, a total of 4,820 participants had data on genotyping and retinal vessel measurements for the current analyses.

A full eye examination was implemented at baseline including taking fundus color transparencies of the optic disc (20^0^ field, Topcon Optical Company, Tokyo, Japan) after pharmacological mydriasis. The transparencies from both eyes were digitized with a high-resolution scanner (Nikon LS-4000, Nikon Corporation, Japan), and the digitized image of one eye with the best quality was analyzed with the Retinal Vessel Measurement System (Retinal Analysis, Optimate, WI; Department of Ophthalmology & Visual Science, University of Wisconsin-Madison). For each participant one summary value was calculated for the arteriolar diameters (CRAE in μm) and one for the venular caliber (CRVE) [2,3]. Pearson’s correlation coefficients for inter-grader agreement were 0.67 – 0.80 for CRAE and 0.91 – 0.94 for CRVE. The corresponding figures were 0.69 – 0.88 and 0.90 – 0.95 for intra-grader agreement.

- - 1. *Blue Mountain Eye Study (BMES)*

The Blue Mountains Eye Study (BMES) is a population-based cohort of a predominantly white population in west of Sydney, Australia. At baseline (1992 – 1994), 3,654 permanent residents aged 49 years or older participated (participation rate of 82.4%) [8]. During 1997 – 1999 (BMES IIA), 2,335 participants (75.1% of survivors) returned for examinations during after 5 years. During 1999 – 2000, 1,174 (85.2%) new participants took part in an Extension Study of the BMES (BMES IIB). BMES cross-section II thus includes BMES IIA (66.5%) and BMES IIB (33.5%) participants (n = 3,509). From the BMES cross section II who had blood samples collected, DNA was extracted for 3,189 (90.1 %) participants.

Stereoscopic retinal photographs (30º) were taken for the macula, optic disc and other retinal fields of both eyes at each visit, using a Zeiss FF3 fundus camera (Carl Zeiss, Oberkochen, Germany) after pupil dilation. The retinal arteriolar and venular calibre was measured from the optic disc-centered photographs taken at baseline examinations, using a validated computer-assisted method [9]. A digitized grid was placed over the image and all vessels passing completely through a zone between 0.5 to 1 disc diameters away from the disc margin were measured, using the Retinal Analysis software (termed Howard program, Optimate, WI; Department of Ophthalmology & Visual Science, University of Wisconsin-Madison). For each vessel and branch, five equidistant measures were taken in μm. Estimates of CRAE and CRVE obtained from the Parr-Hubbard formulas were further converted into estimates equivalent to measures that were based on six largest arterioles and venules respectively of each eye, following the Parr-Hubbard-Knudtson formulas that was used by a newer computer-assisted program (IVAN program) [2,3]. Quadratic weighted kappa values were 0.85 for CRAE and 0.90 for CRVE for inter-grader reliability respectively, and between 0.80-0.93 and 0.80-0.92 for intra-grader reliability of graders 1 and 2, respectively.

Participants of the BMES cross section II who had DNA available were genotyped (N = 2,761) using the Illumina Human 670-Quadv1 custom genotyping array at the Wellcome Trust Centre for Human Genetics, Sanger Institute, Cambridge as part of the Wellcome Trust Case Control Consortium 2 (WTCCC2). A smaller subset (N = 1,356) was also independently genotyped using the Illumina 610-Quad genotyping array at the Hunter Medical Research Institute, Newcastle, Australia, facilitating a detailed assessment of genotyping quality and reproducibility. Principal components (up to four dimensions) were used as covariates in the association analysis.

Imputation was performed using the Hapmap release 24 CEU panel (NCBI Build 36.1) as the imputation backbone, using only single nucleotide polymorphism (SNP) passing quality control (minor allele frequency MAF > 0.01, genotyping call rate > 0.95, Hardy-Weinberg Equilibrium (HWE) *P*-value > 1 x 10^-6^) in both the WTCCC2 and Newcastle datasets. Genotype imputation was performed for autosomal SNPs using Markov Chain Haplotyping (MaCH) package v1.0.16 (<http://www.sph.umich.edu/csg/abecasis/MACH/>). Imputed genotypes were excluded if their ratio of observed to expected variance of imputed allele dosages (R^2^) < 0.3 OR minor allele frequency < 0.01 OR Hardy-Weinberg *P*-value < 1 x 10^-6^.

- 1. Replication cohorts
     1. *Australian Twins Study*

The Australian Twin Eye Study consisted of participants examined as part of the Twins Eye Study in Tasmania (TEST) or the Brisbane Adolescent Twins Study (BATS) [10]. Participants were genotyped on the Illumina Human Hap610W Quad array, with part of the samples genotyped along with the UK Twins Cohort and the rest with DeCODE genetics. Complete phenotype and genetic data were available for 1,709 individuals.

Optic disc photographs were taken with a Nidek 3-Dx simultaneous stereoscopic 15° fundus camera (Nidek, Gamagori, Japan) in all subjects after instillation of tropicamide 1%. Images were developed on colored 35 mm slides (Ektachrome, Eastman Kodak, Rochester, New York) and further digitized (Nikon CoolScan IV ED slide scanner, Nikon Corp., Tokyo, Japan). Retinal vascular caliber was measured using the Parr-Hubbard-Knudtson formulas with computer-assisted software (IVAN, University of Wisconsin) and summarized as CRAE and CRVE [11]. Intra-grader and inter-grader intra-class correlation coefficients were very high for both CRAE (0.95, 0.93) and CRVE (0.99, 0.98).

For genotyping filters, the exclusion criteria for SNPs were MAF ≤ 1%, HWE *P*-value < 10^-6^, SNP call rate ≤ 95% and Illumina Beadstudio Gencall Score < 0.7. Imputation was done against the backbone of Hapmap II release 22 CEU using MaCH2 (<http://www.sph.umich.edu/csg/abecasis/MACH/>) [12]. To account for the relatedness of the individuals, the association analysis was performed using the score test on Merlin (–fastAssoc option) (http://www.sph.umich.edu/csg/abecasis/merlin/) [13].

- - 1. *Multi-Ethnic Study of Atherosclerosis (MESA)*

The Multi-Ethnic Study of Atherosclerosis (MESA) is a study of the characteristics of subclinical cardiovascular disease (disease detected non-invasively before it has produced clinical signs and symptoms) and the risk factors that predict progression to clinically overt cardiovascular disease or progression of the subclinical disease. MESA researchers study a diverse, population-based sample of 6,814 asymptomatic men and women aged 45 – 84 recruited from six field centers across the United States in 2000 and 2001. Among the recruited participants, 38% were white, 28% African-American, 22% Hispanic and 12% Asian, predominantly of Chinese descent [14]. Genome wide association scan (GWAS) and retinal data were available for 2,170 European subjects. Genotyping was performed using the Affymetrix Genome-Wide Human SNP Array 6.0.

Fundus photography was offered to all participants at the second examination. Retinal images were acquired using a standardized protocol and a 45-degree nonmydriatic camera (Canon, Lake Success, New York) [15]. Retinal arteriolar caliber was, measured by the Ocular Epidemiology Reading Center (Madison Wisconsin) and employed the same standardized protocol and software used for the AGES study. The Parr-Hubbard-Knudtson formulas were used to compute summary measures for CRAE and CRVE in micrometer (µm).

IMPUTE version 2.1.0 [16] was used to perform imputation for the MESA European participants (chromosomes 1-22) using HapMap Phase I and II - CEU as the reference panel (release #24 - NCBI Build 36 (dbSNP b126)). SNPs with MAF less than 0.01 or HWE *P*-value < 0.0001 were removed from the analysis. Association tests were performed by SNPTEST v2 [17].

1. **Genotyping quality control filters and imputation of discovery cohorts**

Sample specific quality control filters used in the discovery cohorts included sample failure, sample call rate, sample heterozygosity, excessive Mendelian errors in the samples, outliers identified by IBS clustering analysis and gender discordance [18]. SNP quality control filters included call rates, minor allele frequency, Hardy-Weinberg equilibrium and differential missingness by outcome or genotype [18].

The discovery cohorts were screened for latent population substructure using suitable programs (i.e, EIGENSTRAT in ARIC and AGES [19,20], an identity-by-descent IBD matrix in Rotterdam, principal component analysis in CHS and multi-dimensional scaling in BMES [21]) and found no evidence of occult population admixture. BMES in addition, corrected for the first four principal components in their regression analysis, to retain more samples.

For imputation, high quality SNP data was used as seed for imputation in each study. These criteria included call rates (> 97% in AGES, > 95% in ARIC and CHS, ≥ 95% in BMES, > 98% in RS), MAF filtering of greater than 0.01 in each study, Hardy-Weinberg filtering (HWE *P*-value > 1 x 10^-5^ in CHS and *P*-value > 1 x 10^-6^ in AGES, ARIC, RS and BMES), and differential missingness filter of *P*-value > 1 x 10^-9^ in each study. Genotype imputation for discovery cohorts was performed using the MACH package (<http://www.sph.umich.edu/csg/abecasis/MACH>) [12] (version 1.0.15 for ARIC and RS; v1.0.16 for AGES and BMES) and BIMBAM10 v0.99 [22] for CHS. All the cohorts imputed with the reference panel of Hapmap II CEU release 22 build 36, except CHS which imputed against the reference panel of Hapmap II CEU release 21A build 35. The ratio of the empirically observed dosage variance to the expected binomial dosage variance was estimated as a reliability measure of imputation for each SNP.

We checked bivariate plots of –log10 observed *P*-value against –log10 empirical *P*-value (P-P) plots to ensure that the p-value distribution in each of the discovery cohorts conformed to a null distribution except at the extreme tail. The genomic inflation factor (λ_gc_) measuring the over-dispersion of test-statistics from association tests were 1.040 for AGES, 1.036 for ARIC, 1.041 for CHS, 1.039 for RS and 1.004 for BMES.

1. **Meta-analysis**

Each cohort had approximately 2.5 million SNPs either genotyped or imputed for meta-analysis (2,408,992 for AGES, 2,475,681 for ARIC2, 250,210 for CHS and 2,447,961 for RS and 2,411,691 for BMES). 2,137,729 SNPs were common across all the discovery cohorts and only SNPs that were present in all 5 cohorts were selected for follow-up.

The inverse variance fixed effects meta-analysis method was used for the meta-analysis, with genomic control applied to each cohort prior to meta-analysis. Larger weights were given to studies with bigger samples sizes, directly genotyped SNPs and SNPs with high quality imputation. The meta-analysis was implemented using METAL (<http://www.sph.umich.edu/csg/abecasis/Metal/index.html>). The genomic inflation factor after meta-analysis was 1.022812.

1. **Analyses with Macrovasular Diseases**
   - 1. *Wellcome Trust Case Control Consortium (WTCCC)*

The Wellcome Trust Case Control Consortium (WTCCC) was established to look into the design and analyses of GWA studies and published their landmark paper on the case control analyses of seven common diseases and a set of common controls [17]. The seven diseases included bipolar disorder (BD), coronary artery disease (CAD), Crohn’s disease (CD), hypertension (HT), rheumatoid arthritis (RA), type 1 diabetes (T1D), and type 2 diabetes (T2D). CAD cases were defined as having a validated history of either myocardial infarction or coronary revascularization (coronary artery bypass surgery or percutaneous coronary angioplasty) before their 66^th^ birthday and verified from hospital records or the primary care physician. The control samples were from two sources, the 1958 Birth Cohort and a new UK Blood Service which was established specifically for this study and is a UK national repository of anonymized DNA samples from 3,622 consenting blood donors. Imputation was performed using the Markov Chain Haplotyping (MaCH) package (<http://www.sph.umich.edu/csg/abecasis/MACH>) [12] with Hapmap CEU as the reference panel. Logistic regression was performed using, the estimated dosage of the effective allele from MaCH and the ProbABEL program (<http://mga.bionet.nsc.ru/~yurii/ABEL/>) [23] with no adjustment for covariates.

- - 1. *The Heart and Vascular Health Study (HVH)*

The Heart and Vascular Health Study (HVH) is a population-based case-control study within Group Health (GH) in western Washington State. Myocardial Infarction (MI) cases were GH enrollees, 30 to 79 years of age, who had an incident MI between 1995 and 2006, and alive at the time of study recruitment. Stroke cases were also GH enrollees, 30 to 79 years of age, who had an incident stroke between 1995 and 2008, and alive at the time of study recruitment. Hospital discharge diagnosis codes were used to identify cases and further validated by medical record review as previously described [24]. Control subjects were made up of a stratified random sample of GH enrollees frequency matched to MI cases on age (within decade), sex, hypertension status, and calendar year of identification. If participants were recent enrollees at GH, had a history of prior MI or stroke, or if the incident MI or stroke was a complication of a procedure or surgery, they were excluded from the study. All participants provided written informed consent.

- - 1. *Global Blood Pressure Genetics (Global BPgen) Consortium*

The Global BPgen consortium comprises of 17 individual GWAS studies [25]: the Baltimore Longitudinal Study of Aging (BLSA), British 1958 Birth Cohort (B58C-T1DGC and B58C-WTCCC), Cohorte Lausannoise (CoLaus), Diabetes Genetics Initiative (DGI), European Prospective Investigation of Cancer-Norfolk-Genome Wide Association Study (EPIC-Norfolk-GWAS), Fenland Study, Finland-United States Investigation of NIDDM Genetics (FUSION) study, Invecchiare in Chianti (InCHIANTI), Kooperative Gesundheitsforschung in der Region Augsburg (KORA), the Myocardial Infarction Genetics Consortium (MIGen), Northern Finland Birth Cohort of 1966 (NFBC1966), SardiNIA, Study of Health in Pomerania (SHIP), the Precocious Coronary Artery Disease (PROCARDIS), Supplementation en Vitamines et Mine´raux Antioxydants (SU.VI.MAX) and TwinsUK. Individuals >70 years of age, ascertained on case status for type 1 or 2 diabetes (DGI, FUSION), coronary artery disease (MIgen, PROCARDIS) or hypertension (BRIGHT), were excluded for analysis. Hypertension was defined as either (i) systolic blood pressure ≥ 140 mm Hg or (ii) diastolic blood pressure ≥90 mm Hg or (iii) self-reported hypertension medication. Normotensive controls were defined as individuals not taking any antihypertensives and having a systolic blood pressure ≤ 120 mm Hg and a diastolic blood pressure ≤ 85 mm Hg. Analysis of hypertension was done using logistic regression to adjust for age, age^2^, sex and body mass index (BMI) for 34,433 individuals.

- - 1. *Genetics of Diabetes and Audit Research in Tayside Scotland (GoDARTS)*

GoDARTS is a population based case control study in Tayside, Scotland to study the epidemiology and genetics of Type 2 Diabetes [26]. Participants were recruited between 2004 to 2009 and linked to routinely collected medical records available for the Tayside region. Participants with coronary artery disease (CAD) were identified from medical records. These included hospital admissions and deaths due to myocardial infarction and unstable angina, and participants who underwent coronary revascularization. Stroke was defined as hospital admissions and deaths associated with ischemic stroke.

Quality controlled genotyping data for the SNPs of interest were available for 3,328 diabetics genotyped on the Affymetrix Genome-Wide Human SNP Array 6.0.

A multivariate Cox regression analysis was performed on incident CAD and incident ischemic stroke using the survival package in R (<http://CRAN.R-project.org/package=survival>) [27]. Incident events were defined as events that occurred after participants were recruited into the study. They were followed up until their first stroke or CAD event or death from other causes or until the end of the censoring period. Baseline measures were recorded at the time of recruitment. An additive mode was used where the effect allele was coded as the allele that reduced arteriolar caliber with age, gender, body mass index, history of smoking, previous coronary artery disease/stroke events at baseline included as covariates in the analysis.

A multivariable logistic regression analysis was also performed in prevalent cases of CAD in GoDARTS, corrected for age, gender, BMI, history of smoking and use of hypertension medication.

1. **References**

1. Harris TB, Launer LJ, Eiriksdottir G, Kjartansson O, Jonsson PV, et al. (2007) Age, Gene/Environment Susceptibility-Reykjavik Study: multidisciplinary applied phenomics. Am J Epidemiol 165: 1076-1087.

2. Hubbard LD, Brothers RJ, King WN, Clegg LX, Klein R, et al. (1999) Methods for evaluation of retinal microvascular abnormalities associated with hypertension/sclerosis in the Atherosclerosis Risk in Communities Study. Ophthalmology 106: 2269-2280.

3. Knudtson MD, Lee KE, Hubbard LD, Wong TY, Klein R, et al. (2003) Revised formulas for summarizing retinal vessel diameters. Curr Eye Res 27: 143-149.

4. Qiu C, Cotch MF, Sigurdsson S, Garcia M, Klein R, et al. (2008) Retinal and cerebral microvascular signs and diabetes: the age, gene/environment susceptibility-Reykjavik study. Diabetes 57: 1645-1650.

5. (1989) The Atherosclerosis Risk in Communities (ARIC) Study: design and objectives. The ARIC investigators. Am J Epidemiol 129: 687-702.

6. Fried LP, Borhani NO, Enright P, Furberg CD, Gardin JM, et al. (1991) The Cardiovascular Health Study: design and rationale. Ann Epidemiol 1: 263-276.

7. Hofman A, Breteler MM, van Duijn CM, Janssen HL, Krestin GP, et al. (2009) The Rotterdam Study: 2010 objectives and design update. Eur J Epidemiol 24: 553-572.

8. Mitchell P, Smith W, Attebo K, Wang JJ (1995) Prevalence of age-related maculopathy in Australia. The Blue Mountains Eye Study. Ophthalmology 102: 1450-1460.

9. Leung H, Wang JJ, Rochtchina E, Tan AG, Wong TY, et al. (2003) Relationships between age, blood pressure, and retinal vessel diameters in an older population. Invest Ophthalmol Vis Sci 44: 2900-2904.

10. Mackey DA, Mackinnon JR, Brown SA, Kearns LS, Ruddle JB, et al. (2009) Twins eye study in Tasmania (TEST): rationale and methodology to recruit and examine twins. Twin Res Hum Genet 12: 441-454.

11. Sun C, Zhu G, Wong TY, Hewitt AW, Ruddle JB, et al. (2009) Quantitative genetic analysis of the retinal vascular caliber: the Australian Twins Eye Study. Hypertension 54: 788-795.

12. Li Y, Willer CJ, Ding J, Scheet P, Abecasis GR (2010) MaCH: using sequence and genotype data to estimate haplotypes and unobserved genotypes. Genet Epidemiol 34: 816-834.

13. Abecasis GR, Cherny SS, Cookson WO, Cardon LR (2002) Merlin--rapid analysis of dense genetic maps using sparse gene flow trees. Nat Genet 30: 97-101.

14. Bild DE, Bluemke DA, Burke GL, Detrano R, Diez Roux AV, et al. (2002) Multi-ethnic study of atherosclerosis: objectives and design. Am J Epidemiol 156: 871-881.

15. Klein R, Klein BE, Knudtson MD, Wong TY, Cotch MF, et al. (2006) Prevalence of age-related macular degeneration in 4 racial/ethnic groups in the multi-ethnic study of atherosclerosis. Ophthalmology 113: 373-380.

16. Marchini J, Howie B, Myers S, McVean G, Donnelly P (2007) A new multipoint method for genome-wide association studies by imputation of genotypes. Nat Genet 39: 906-913.

17. (2007) Genome-wide association study of 14,000 cases of seven common diseases and 3,000 shared controls. Nature 447: 661-678.

18. Psaty BM, O'Donnell CJ, Gudnason V, Lunetta KL, Folsom AR, et al. (2009) Cohorts for Heart and Aging Research in Genomic Epidemiology (CHARGE) Consortium: Design of prospective meta-analyses of genome-wide association studies from 5 cohorts. Circ Cardiovasc Genet 2: 73-80.

19. Price AL, Patterson NJ, Plenge RM, Weinblatt ME, Shadick NA, et al. (2006) Principal components analysis corrects for stratification in genome-wide association studies. Nat Genet 38: 904-909.

20. Patterson N, Price AL, Reich D (2006) Population structure and eigenanalysis. PLoS Genet 2: e190.

21. Purcell S, Neale B, Todd-Brown K, Thomas L, Ferreira MA, et al. (2007) PLINK: a tool set for whole-genome association and population-based linkage analyses. Am J Hum Genet 81: 559-575.

22. Servin B, Stephens M (2007) Imputation-based analysis of association studies: candidate regions and quantitative traits. PLoS Genet 3: e114.

23. Aulchenko YS, Struchalin MV, van Duijn CM (2010) ProbABEL package for genome-wide association analysis of imputed data. BMC Bioinformatics 11: 134.

24. Psaty BM, Heckbert SR, Koepsell TD, Siscovick DS, Raghunathan TE, et al. (1995) The risk of myocardial infarction associated with antihypertensive drug therapies. JAMA 274: 620-625.

25. Newton-Cheh C, Johnson T, Gateva V, Tobin MD, Bochud M, et al. (2009) Genome-wide association study identifies eight loci associated with blood pressure. Nat Genet.

26. Morris AD, Boyle DI, MacAlpine R, Emslie-Smith A, Jung RT, et al. (1997) The diabetes audit and research in Tayside Scotland (DARTS) study: electronic record linkage to create a diabetes register. DARTS/MEMO Collaboration. BMJ 315: 524-528.

27. Therneau T (2011) Survival: Survival analysis, including penalised likelihood. 2.36-9 ed.
